# The landscape of chromothripsis across adult cancer types

**DOI:** 10.1038/s41467-020-16134-7

**Published:** 2020-05-08

**Authors:** Natalia Voronina, John K. L. Wong, Daniel Hübschmann, Mario Hlevnjak, Sebastian Uhrig, Christoph E. Heilig, Peter Horak, Simon Kreutzfeldt, Andreas Mock, Albrecht Stenzinger, Barbara Hutter, Martina Fröhlich, Benedikt Brors, Arne Jahn, Barbara Klink, Laura Gieldon, Lina Sieverling, Lars Feuerbach, Priya Chudasama, Katja Beck, Matthias Kroiss, Christoph Heining, Lino Möhrmann, Andrea Fischer, Evelin Schröck, Hanno Glimm, Marc Zapatka, Peter Lichter, Stefan Fröhling, Aurélie Ernst

**Affiliations:** 1 Group Genome Instability in Tumors, DKFZ, Heidelberg, Germany; 2 Division of Molecular Genetics, DKFZ, Heidelberg, Germany; 3 German Cancer Consortium (DKTK), Heidelberg, Germany; 4 Computational Oncology, Molecular Diagnostics Program, National Center for Tumor Diseases, DKFZ, Heidelberg, Germany; 5 Heidelberg Institute for Stem cell Technology and Experimental Medicine (HI-STEM), Heidelberg, Germany; 6 Department of Pediatric Immunology, Hematology and Oncology, Heidelberg University Hospital, Heidelberg, Germany; 7 Division of Applied Bioinformatics, DKFZ and NCT Heidelberg, Heidelberg, Germany; 8 Faculty of Biosciences, Heidelberg University, Heidelberg, Germany; 9 Division of Translational Medical Oncology, National Center for Tumor Diseases (NCT) Heidelberg and DKFZ, Heidelberg, Germany; 10 DKFZ-Heidelberg Center for Personalized Oncology (HIPO), Heidelberg, Germany; 11 Institute of Pathology, Heidelberg University Hospital, Heidelberg, Germany; 12 Institute for Clinical Genetics, Faculty of Medicine Carl Gustav Carus, TU Dresden, ERN-GENTURIS, Hereditary Cancer Syndrome Center Dresden, German Cancer Consortium (DKTK), Dresden, Germany; 13 German Cancer Research Center (DKFZ), Heidelberg, Germany; 14 National Center for Tumor Diseases (NCT), Dresden, Germany; 15 Department of Translational Medical Oncology, NCT Dresden, Dresden, and DKFZ, Dresden, Germany; 16 Center for Personalized Oncology, University Hospital Carl Gustav Carus Dresden, Technical University of Dresden, Dresden, Germany; 17 German Cancer Consortium (DKTK), Dresden, Germany; 18 National Center of Genetics (NCG), Laboratoire national de santé (LNS), Dudelange, Luxembourg; 19 Precision Sarcoma Research Group, DKFZ, National Center for Tumor (NCT) Diseases, Heidelberg, Germany; 20 Department of Internal Medicine I, Division of Endocrinology and Diabetology, University Hospital Würzburg, Würzburg, Germany

**Keywords:** Cancer genomics, Cancer genetics

## Abstract

Chromothripsis is a recently identified mutational phenomenon, by which a presumably single catastrophic event generates extensive genomic rearrangements of one or a few chromosome(s). Considered as an early event in tumour development, this form of genome instability plays a prominent role in tumour onset. Chromothripsis prevalence might have been underestimated when using low-resolution methods, and pan-cancer studies based on sequencing are rare. Here we analyse chromothripsis in 28 tumour types covering all major adult cancers (634 tumours, 316 whole-genome and 318 whole-exome sequences). We show that chromothripsis affects a substantial proportion of human cancers, with a prevalence of 49% across all cases. Chromothripsis generates entity-specific genomic alterations driving tumour development, including clinically relevant druggable fusions. Chromothripsis is linked with specific telomere patterns and univocal mutational signatures in distinct tumour entities. Longitudinal analysis of chromothriptic patterns in 24 matched tumour pairs reveals insights in the clonal evolution of tumours with chromothripsis.

## Abstract (web summary)

The shattering of chromosomes is a dramatic early event in tumourigenesis and is termed chromothripsis. Here, the authors examine chromothripsis across 28 tumour types and show that 49% of cancers exhibit features of chromothripsis.

## Introduction

The development of next-generation sequencing technologies and their applications in cancer genome studies have enabled the discovery of a new form of genome instability called chromothripsis^1,2^. This catastrophic process drastically contrasts with the classical view of multi-step tumour evolution. In a presumably single event, chromothripsis leads to extensive chromosome rearrangements, fostering the simultaneous acquisition of multiple genomic aberrations^3^. Following the partial or full shattering of one or a few chromosome(s) via tens to hundreds of DNA double-strand breaks, imperfect repair occurs. DNA fragments not reincorporated into the derivative chromosome are often lost to the cell. Within one or very few cell cycles, tumour suppressor functions are disrupted and/or oncogenic fusions and oncogene amplifications arise^3^. Cells that survive such a cataclysm likely have gained a strong selection advantage due to their massively rearranged genome - a phenomenon that potentially transforms such cells into cancer cells. Importantly, chromothripsis is linked with aggressive tumour behaviour and poor prognosis for cancer patients^2,4–6^.

Despite initial prevalence estimates in the range of two to three per cent of all cancer cases^1^, chromothripsis is probably much more widespread than originally suspected. As more cancer genomes are being sequenced, more cancer types with high frequencies of chromothriptic events emerge^7–10^, suggesting that chromothripsis might play a major role in a substantial number of human cancers. The prevalence varies from zero to 100% across tumour (sub)entities^11^. However, comparisons between studies and between tumour entities are challenging for several reasons. First, the type of data used for chromothripsis scoring differ, with high-coverage whole-genome sequencing providing the most reliable scoring, but other methods offering lower resolution also being employed (e.g., SNP arrays or array comparative genomic hybridization). Second, varying definitions and minimal criteria for inferring chromothripsis have been applied, from relatively loose to conservative cutoff values^3^. Third, visual versus automated scoring both have advantages and weaknesses, with risks of false-positive and false-negative cases for each of the methods. Ideally, automated scoring and manual review used together offer the most reliable chromothripsis inference, as reported in a recent study on melanoma^12^.

To ensure the best comparability across tumour entities, chromothripsis scoring needs to be performed with a standardized workflow. Three previous studies described pan-cancer analyses of chromothripsis. Cai and colleagues performed a comprehensive analysis of chromothriptic-like patterns in large SNP array and comparative genomic hybridization datasets^13^. However, this type of data, even though available for large cohorts, does not allow formal testing of all criteria defining chromothripsis for a conclusive identification of bona fide chromothriptic cases^3^. In the second study, Gröbner and colleagues analysed chromothripsis in paediatric cancer^14^, focusing on tumour entities that are frequent in children. Cortés-Ciriano and colleagues performed automated scoring of chromothripsis in cancer genomes from the PCAWG consortium^15^ (see discussion for a comparison of the chromothripsis prevalences between studies).

Here we present a comprehensive sequencing-based analysis of chromothripsis in a pan-cancer cohort of 634 adult tumours comprising 28 histologic cancer types, including rare tumour entities. Using a standardized workflow, we detect a surprisingly high frequency of chromothriptic events and identify marked differences in genomic features between tumours with or without chromothripsis.

## Results

### Scoring chromothripsis in cancer genomes

We used paired-end Illumina-based sequencing data for 634 tumours from the NCT/DKTK MASTER program (Molecularly Aided Stratification for Tumour Eradication^16^) including 316 whole-genome sequences (WGS, median coverage 80×) and 318 whole-exome sequences (WES, 124×). Tumour and matched germline samples were processed with standardized pipelines to detect single nucleotide variants (SNVs), short insertions and deletions (indels), copy-number variants (CNVs) and other structural variants. The full tumour cohort is described in Supplementary data 1.

**Fig. 1:**
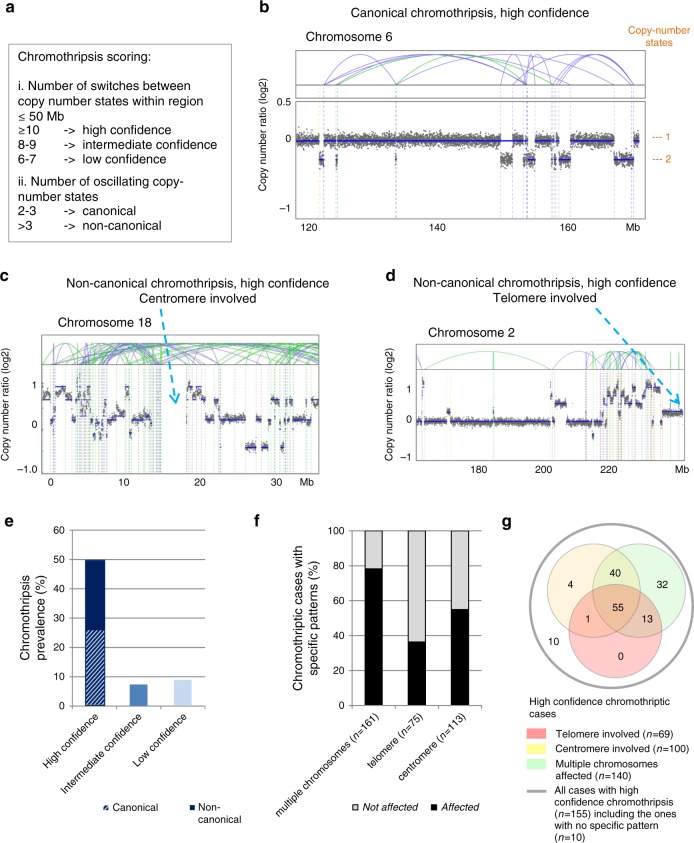
Chromothriptic patterns and prevalence in 316 whole-genome sequences. **a** Chromothripsis scoring: criteria to determine the confidence of the scoring and to define canonical versus non-canonical chromothripsis. **b** Representative example of canonical chromothripsis. **c** Representative example of non-canonical chromothripsis. In this case, the centromere is included in the chromothriptic region. **d** Representative example of chromothriptic chromosome for which the telomere region is involved. **e** Chromothripsis prevalence shown as percentage of cases (*n* = 316, whole-genome sequencing) including cases with high confidence, intermediate confidence and low confidence chromothripsis. For tumours with high confidence chromothripsis, we distinguish between canonical and non-canonical chromothriptic patterns. **f** Chromothripsis cases with multiple versus single chromosomes affected; with or without telomere involvement (illustrated in **d**), as well as with or without centromere involvement (illustrated in **c**) from all 316 cases. High confidence, intermediate confidence and low confidence chromothriptic cases are shown. **g** Venn diagram showing the overlapping fractions of high confidence chromothriptic cases with multiple chromosomes affected, with or without involvement of telomeres and/or centromeres. Analyses based on whole-exome sequences and examples showing the counting of switches between copy-number states are shown in Supplementary Figs. 1–2. On the copy-number plots, blue lines indicate inversions, green lines indicate break ends, brown lines indicate translocations and orange lines indicate copy-number variation.

We applied established criteria for inferring chromothripsis in cancer genomes^3^ (e.g., ≥10 changes in copy-number on an individual chromosome, see Methods for all details on the scoring). We distinguished canonical chromothripsis involving three or fewer copy-number states from non-canonical chromothripsis involving more than three copy-number states (Fig. 1a–d and Supplementary Fig. 1a–d). To ensure stringent criteria regarding the clustering of the breakpoints, we required at least 10 changes in segmental copy-number within 50 Mb for high-confidence scoring. We confirmed the performance of our chromothripsis scoring by comparing visual scoring and algorithm-based scoring, with a validation rate of 85% (matching scores between both methods, see Supplementary data 2). This combined scoring confirmed the hallmarks of chromothripsis, including clustering of breakpoints and randomness of fragment order and orientation, as defined by Korbel and Campbell^3^. In addition to cases scored positive for chromothripsis with high-confidence (155 of the 316 whole-genome sequences), we also scored intermediate and low-confidence chromothriptic events, with 8–9 and 6–7 switches between copy-number states, respectively.

In parallel, we examined a second group of patients belonging to the same cohort but analysed independently, for which we scored chromothripsis based on whole-exome sequences (*n* = 318), making use of the off-target reads to maximize the resolution of the CNV calling (see Methods). A similar percentage of cases showed rearrangements consistent with chromothripsis as in the tumours scored with available whole-genome sequences (see Methods for details on the scoring criteria based on whole-exome sequences, Supplementary Figs. 1 and 2 and Supplementary Data 1). In addition, we analysed as a validation cohort 18 cases for which we performed both whole-genome and whole-exome sequencing. Scoring chromothripsis independently in these two datasets confirmed our approach, with a concordant scoring status for 16 of the 18 cases despite the lower sensitivity of whole-exome sequencing as compared to whole-genome sequencing. As not all formal criteria defining chromothripsis can be tested based on whole-exome sequencing data (e.g., randomness of DNA fragment joins, “walking” the derivative chromosome^3^), we analysed the whole-genome and whole-exome cohorts separately. Unless otherwise specified, the results below focus on the high-confidence scoring in whole-genome sequences, with the detailed analyses of the whole-exome sequences shown in the Supplementary Data.

### Chromothripsis is common in many cancer entities

**Fig. 2:**
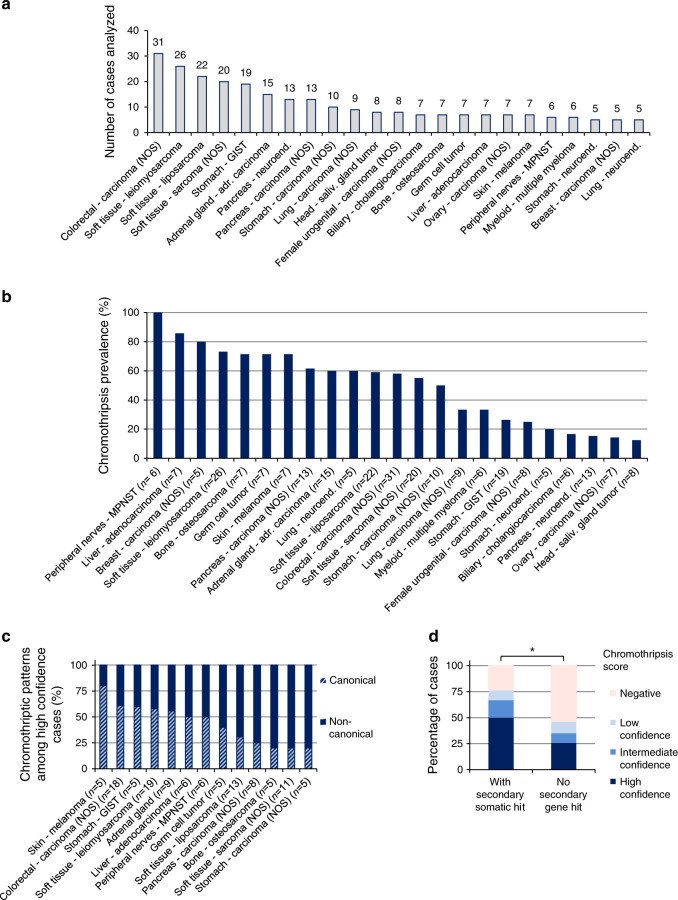
Tumour entities represented in the cohort and chromothripsis prevalence across entities. **a** Number of analysed cases for each tumour entity (*n* = 316 whole-genome sequences in total, with 263 cases for entities with at least 5 cases per entity). Tumour entities with less than 5 cases per entity are shown in Supplementary data 1. **b** Chromothripsis prevalence (high confidence) per tumour entity for all entities with at least 5 cases (*n* = 263). **c** Distribution of canonical versus non-canonical chromothripsis, from all cases with high confidence chromothripsis scoring (*n* indicates the number of high confidence chromothriptic cases per tumour entity for all entities with at least 5 high confidence cases). **d** Chromothripsis prevalence among cases with germline mutations in cancer predisposition genes (*n* = 74 cases, whole-genome and whole-exome sequences). Statistical significance was tested using Fisher exact test (*p < 0.05, two-sided). MPNST malignant peripheral nerve sheath tumour; GIST gastrointestinal stromal tumour; NOS not otherwise specified.

We detected chromothripsis in 49% of all cases (*n* = 316 cases with available whole-genome sequencing data, high-confidence scoring, see Fig. 1e). This prevalence is probably higher than the general prevalence for chromothripsis in cancer for two reasons. First, this cohort has an overrepresentation of specific tumour entities such as sarcomas, which show a high prevalence for chromothripsis^1,10^ (Fig. 2a, b). Second, this cohort includes a vast majority of advanced disease patients, and there is a link between chromothripsis and aggressive tumours^11^. Importantly, for those entities for which previous studies describing chromothripsis existed, our scoring confirms the reported prevalence ranges. For instance, we detected chromothripsis in 33% of the multiple myeloma cases, in line with a previous study reporting a chromothripsis prevalence of 36%^17^. Remarkably, we identified novel tumour entities for which chromothripsis was (i) not described before, despite the high prevalence of this phenomenon in these entities, or (ii) only reported based on analyses other than whole-genome sequencing (e.g., SNP arrays or array comparative genomic hybridization). For instance, 100% of the cases showed chromothripsis in malignant peripheral nerve sheath tumours (MPNSTs, *n* = 6). In germ cell tumours, 71% of the cases showed chromothripsis (*n* = 9). Therefore, chromothripsis is apparently a key initiating event in a number of tumour entities for which this phenomenon was not suspected to play a major role.

Furthermore, we observed distinct patterns of chromothriptic events across tumour entities (Fig. 1f,g). Notably, the majority of tumours with chromothripsis harboured several chromothriptic chromosomes (78% of the chromothriptic events). Leiomyosarcomas, liposarcomas and osteosarcomas frequently showed more than three chromosomes affected by chromothripsis (see Supplementary data 1). Conversely, gastrointestinal stromal tumours and pancreas carcinomas typically had only one chromosome per tumour affected by chromothripsis. The existence of different chromothriptic patterns suggest different mechanisms leading to chromothripsis across tumour entities, and possibly among cases within one given entity. Among other mechanisms, telomere dysfunction and DNA damage in micronuclei (i.e. abnormal nuclear structures containing one or a few chromosomes or acentric chromosome fragments) have been proposed to lead to chromothripsis^18–22^. In the majority of our cases (64%), the telomere region was not affected by the chromothriptic event. However, chromothriptic events that do not directly affect telomere regions can also result from telomere fusions, since the genomic regions included in chromatin bridges can be distant from the telomeres, depending on the structure of the dicentric chromosomes formed in telomere crisis^18^. In approximately half of the tumours (55%), the centromere was included in the segment affected by chromothripsis. Altogether, associations between chromothripsis and distinct genomic features support pan-cancer and entity-specific mechanisms leading to chromothripsis.

### Germline variants linked with chromothripsis

Germline mutations in *TP53* and in *ATM* are strongly linked with chromothripsis^23,24^, suggesting that inactivation of essential checkpoints or DNA repair factors may facilitate chromothripsis occurrence. With the exception of one *ATM* mutation carrier for which no somatic loss of the wild-type allele was detected, germline mutations in *ATM* and *TP53* were also tightly linked with chromothripsis in this cohort. To find novel germline variants associated with chromothripsis, we systematically assessed pathogenic germline variants across a set of autosomal cancer predisposition genes. We identified pathogenic germline variants across cancer predisposition genes including among others DNA repair genes from mismatch repair (*MSH2, MSH6, MLH1*) and double-strand break repair (*ATM, NBN, BRCA1, BRCA2*). Approximately 40% of the tumours with germline variants exhibited somatic loss of the wild-type allele. Importantly, the chromothripsis prevalence was significantly higher in tumours with somatic loss of the wild-type allele as compared to cases for which no secondary hit was detected (high-confidence scoring: 50% versus 25.6%, respectively, see Fig. 2d). Therefore, loss of the wild-type allele in these genes likely facilitates chromothripsis occurrence. For a subset of the cancer predisposition genes, such as *SDHA* and *SDHB* (encoding for mitochondrial enzymes), none of the tumours showed chromothripsis, despite a secondary somatic hit in all cases (*n* = 5). Therefore, this approach may help to identify genes whose inactivation facilitates chromothripsis occurrence.

### Chromothripsis generates entity-specific cancer drivers

**Fig. 3:**
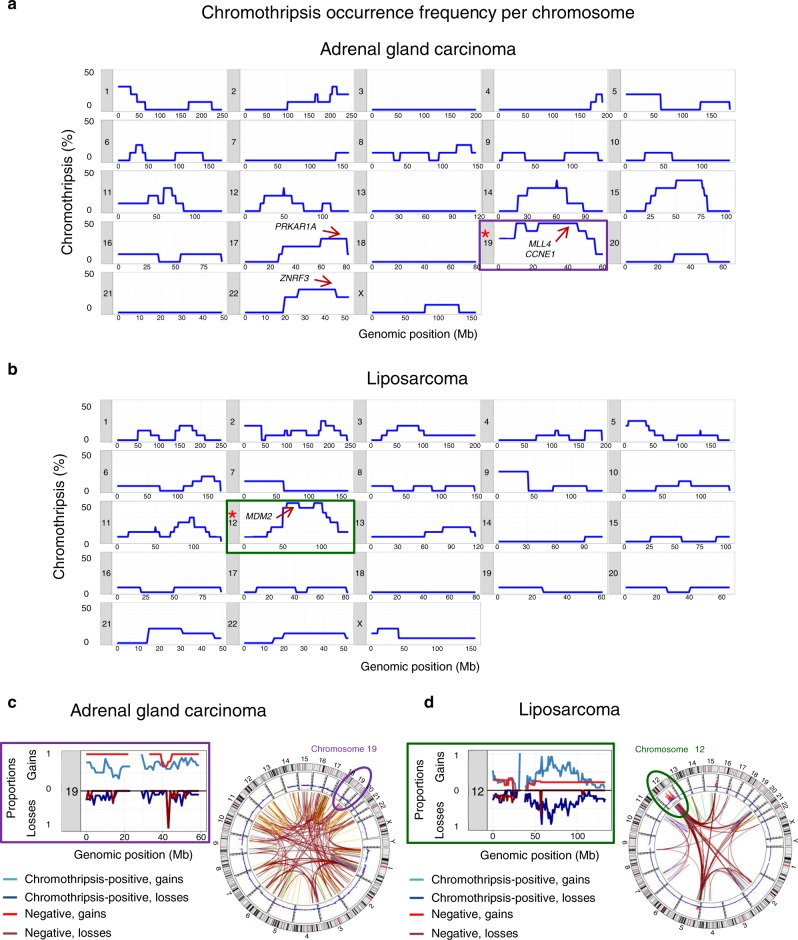
Frequency of chromothriptic events across chromosomes for two representative tumour entities. **a** adrenal gland adenocarcinoma, *n* = 15 cases; **b** liposarcoma, *n* = 13 cases, total for high and intermediate confidence scoring. The Y axis shows the percentage of chromothriptic events affecting each chromosomal fragment from all chromothriptic cases. Location of known driver genes frequently affected by chromothriptic events is indicated by arrows. Stars indicate chromosomes that are significantly enriched for chromothriptic events in these tumour entities (permutation test, see also Supplementary data 3). For the frequencies of chromothriptic events on all chromosomes in other tumour entities, please refer to Supplementary Fig. 3. **c**,**d** Proportions of gains (upper panels) and losses (lower panels) in tumours with chromothripsis (blue) or without chromothripsis (red) for representative chromosomes frequently affected by chromothripsis, with one illustrative CIRCOS plot for each tumour entity. Lines on CIRCOS plots show deletions in green, translocations in brown, duplications in orange and inversions in blue.

Chromothripsis promotes cancer development by disrupting tumour suppressor genes and by activating oncogenes^1,2,11^. For each tumour entity, we identified specific chromosomes and chromosome regions significantly more frequently affected by chromothriptic events than expected by chance (permutation test, Fig. 3 and Supplementary Fig. 3; see Supplementary data 3 for *p* values associated with the enrichment of specific chromosomes in each tumour type). We hypothesize that this unequal distribution does not originate from a more frequent occurrence of chromothripsis on specific chromosomes in a given cell type in the first place, but rather from the selection advantage provided by a chromothriptic event when cell type specific drivers are affected. For instance, in adrenal gland adenocarcinomas, chromothriptic events are predominantly detected on chromosomes 17, 19 and 22, affecting driver genes known to play an essential role in this tumour entity, such as *PRKAR1A, MLL4, CCNE1* and *ZNRF3*, respectively (Fig. 3). Chromosome regions frequently gained or lost in tumours with chromothripsis were also frequently gained or lost in tumours without chromothripsis, respectively (Fig. 3c,d and Supplementary Fig. 3). This suggests that different processes (chromothripsis or alternative events) alter the copy-number landscape in a non-random fashion by providing selective advantages to the affected cells.

**Fig. 4:**
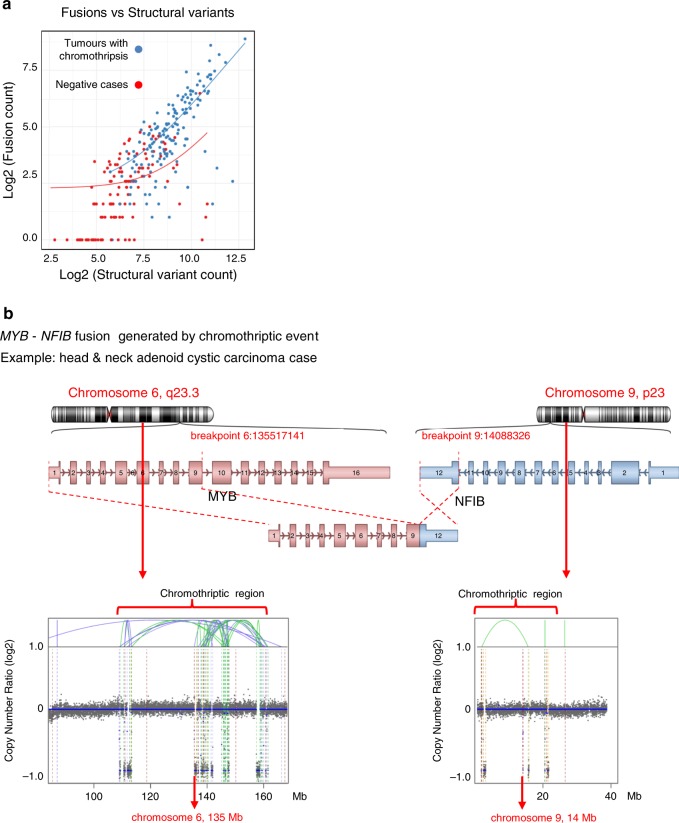
Gene fusions generated by chromothriptic events. **a** Tumours with chromothripsis have more gene fusions per structural variant. Regression analysis for the number of fusions depending on the number of structural variants in tumours with or without chromothripsis (see Supplementary data 4 for all parameters of the regression analyses). **b** Chromothripsis generates clinically relevant gene fusions, as illustrated with the *MYB* – *NFIB* fusion, which drives tumour development in adenoid cystic carcinoma. Blue lines indicate inversions, green lines indicate break ends, brown lines indicate translocations and orange lines indicate copy-number variation.

To investigate whether chromothripsis generates clinically relevant gene fusions, we identified fusion transcripts from RNA sequencing. To circumvent issues arising from the reliability of fusion gene predictions, we only considered gene fusions detected with high confidence and with supporting reads from the matching DNA sequencing data. This analysis revealed significantly more fusion transcripts in tumours with chromothripsis as compared to tumours without chromothripsis (Fig. 4a, Supplementary Fig. 4 and Supplementary Data 4). Regression analysis showed that the increased number of fusions in tumours with chromothripsis is not simply due to the number of structural variants but also to the chromothripsis status itself, with five times more fusion genes in tumours with chromothripsis for a given number of structural variants (Fig. 4a and Supplementary Data 4). This finding is highly relevant for the search for druggable targets in tumours with chromothripsis, as a number of fusion genes offer druggable events or diagnostic markers. Notably, we identified the highly oncogenic *MYB–NFIB* fusion, generated by a chromothriptic event (Fig. 4b), which is an important diagnostic marker in head and neck adenoid cystic carcinoma^25,26^.

### Chromothripsis is linked with poor clinical outcome

Chromothripsis was previously linked with poor outcome in several tumour entities, such as medulloblastoma^23^, neuroblastoma^6^ and acute myeloid leukaemia^4^. Several characteristics of this cohort make a survival analysis challenging. First, after sub-dividing all cases by tumour entity, the statistical power within each entity is limited. Second, this cohort is enriched for advanced disease patients, due to the patient selection in the NCT/DKTK MASTER program. Despite these issues, we identified a significant association between chromothripsis and poor outcome (shorter overall survival) in colorectal cancer, which is the tumour entity with the largest number of cases in this cohort (*n* = 33, Supplementary Fig. 5). Importantly, all patients included in this analysis were advanced (metastatic) colorectal cancer patients, with no significant age difference between both groups, further highlighting the importance of the chromothripsis status.

### Distinct telomere patterns in tumours with chromothripsis

Telomere attrition and breakage-fusion-bridge cycles, which lead to dicentric chromosomes and chromosome bridges, were shown to initiate chromothriptic events in leukaemia and in cell culture models^18–20,27^. To investigate the role of telomere dysfunction in chromothripsis, we analysed telomere stabilization mechanisms in tumours with or without chromothripsis. In about 85% of human cancers, telomerase is up-regulated by *TERT* amplifications^28^, rearrangements^29^ or mutations in the *TERT* promoter^30^. The remaining tumours use a repair-based pathway called alternative lengthening of telomeres (ALT), a mechanism based on DNA recombination of telomeric sequences^31^.

**Fig. 5:**
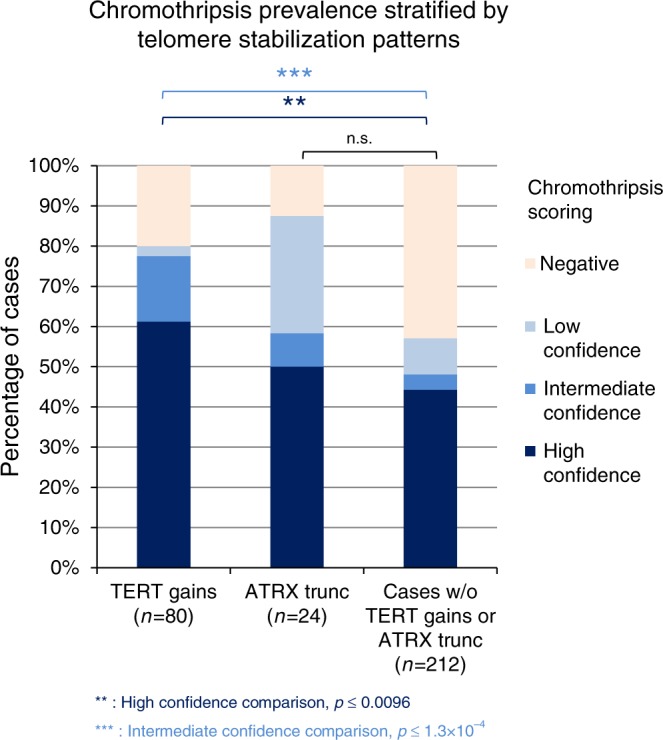
Chromothripsis is associated with specific telomere features. Cases with gains of *TERT* (*n* = 80) or with truncating mutations in *ATRX* (*n* = 24, *ATRX* being strongly linked with activation of the Alternative Lengthening of Telomeres pathway) are enriched in chromothripsis-positive tumours as compared to tumours without *TERT* gain and without *ATRX* mutation (*n* = 212). Statistical analysis was tested using chi squared tests to compare the proportions of tumours with chromothripsis (high-confidence or intermediate-confidence). *ATRX* trunc, tumours with truncating mutations in *ATRX*.

Tumours harbouring *TERT* gains (*n* = 80) showed a significantly higher prevalence for chromothripsis (61%, as compared to 44% in tumours without *TERT* gains, *p* < 0.01, chi square tests, see Fig. 5). In about 9% of the cases with *TERT* gains and chromothripsis, the chromothriptic event was likely the cause of the *TERT* gain, with the *TERT* locus included in the chromothriptic region. Conversely, cases with mutations in the *TERT* promoter were not enriched for chromothriptic events. Truncating mutations in *ATRX* or *DAXX*, which are strongly linked with ALT activation, were not significantly linked with chromothripsis. However, tumours with truncating mutations in *ATRX* or *DAXX* showed the highest proportion of low-confidence chromothriptic events, suggesting a potential enrichment for a form of chromothripsis with few breakpoints in this group. Across all entities, the telomere content was not significantly different between cases with or without chromothripsis. As telomerase upregulation is linked with shorter telomeres, but ALT activation goes along with an increase in the average telomere length, it is possible that differences in telomere length between chromothripsis-positive and chromothripsis-negative cases may be masked, when considering tumours with different telomere stabilization mechanisms. We also analysed structural variants affecting the *TERT* locus, and in particular links between *TERT* and chromothriptic chromosomes. Interestingly, 20% of cases with *TERT* gains showed a structural variant linking *TERT* with one of the chromothriptic chromosomes, whereas only 6% of the cases without *TERT* gain harboured a structural variant between *TERT* and regions affected by chromothripsis (*p* = 0.02, Chi-squared test, see Supplementary Table 1). Altogether, telomere features are strongly linked with chromothripsis, with entity-specific patterns.

### DNA repair processes active in tumours with chromothripsis

**Fig. 6:**
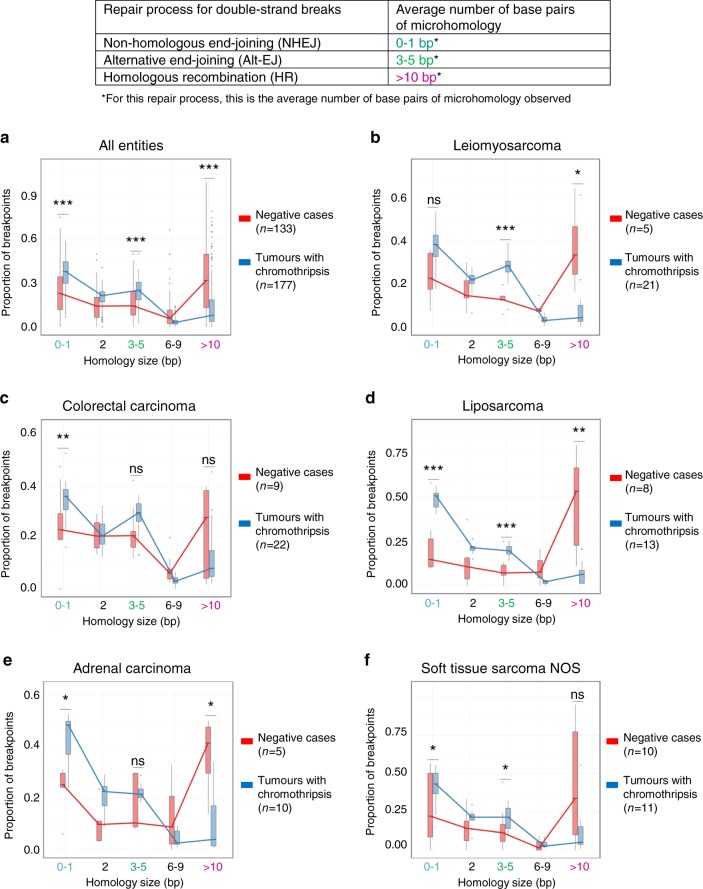
Major DNA repair processes involved in the rejoining of the breakpoints after chromothripsis. Based on the number of base pairs of homology at the breakpoint sites, we can infer the prevailing repair processes involved in the rejoining of the DNA fragments (**a**–**f**). Statistical significance was tested using beta-regression analyses. Family-wise correction of *p* values was performed according to Bonferroni for all tumour entities with at least 15 cases in total, of which at least 5 tumours showed chromothripsis and at least 5 were negative. The comparisons shown here were performed case wise (tumours with versus without chromothripsis). Comparisons performed region wise (chromothriptic chromosomes versus non-chromothriptic chromosomes) are shown in Supplementary Fig. 6. Centre lines show median values, bounds of boxes show 75th percentiles and whiskers show maximum and minimum. (*p < 0.05; **p < 0.01; ***p < 0.001).

Analysing the precise DNA sequence at the chromosome breakpoints allows inferring which repair processes were likely involved in the re-joining of the segments. In a subset of tumour entities, we did not observe any marked difference in terms of homology at the breakpoints between tumours with and without chromothripsis. In such tumour entities, similar repair processes may be involved in the repair of double-strand breaks due to chromothripsis or due to other events. However, in a number of tumour types (e.g., liposarcoma, leiomyosarcoma) blunt ends and short microhomologies (1 bp), most common after repair by non-homologous end-joining, as well as microhomologies of 3–5 bp, frequent after repair by alternative end-joining, were significantly more enriched in tumours with chromothripsis (Fig. 6 and Supplementary Fig. 6). These differences in microhomology length were significant when comparing tumours with versus without chromothripsis (case wise) but also when comparing breakpoints on chromothriptic chromosomes versus the rest of the genome (region wise). Conversely, long homologies (>10 bp) characteristic of repair by homologous recombination were significantly less frequent in tumours with chromothripsis. This is in line with the link between chromothripsis and homologous recombination deficiency that we reported previously^14,32^ and highlights the role of non-homologous end-joining and alternative end-joining in the re-joining of chromothriptic chromosomes.

### Mutational signatures associated with chromothripsis

**Fig. 7:**
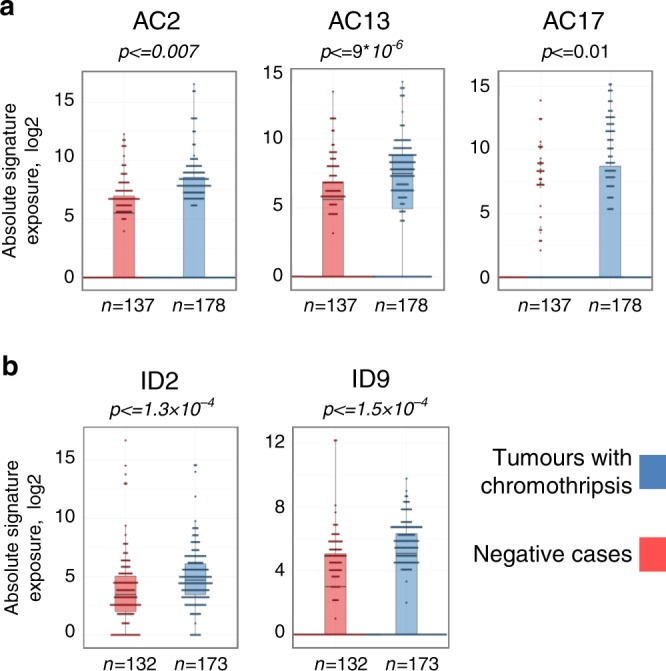
Chromothripsis is linked with specific mutational signatures. Representative examples of base substitution signatures (**a**) and indel signatures (**b**) in chromothripsis-positive versus chromothripsis-negative tumours across all entities. Significance was tested using Wilcoxon tests (two-sided). Family-wise correction of *p* values was performed according to Bonferroni. Supplementary Fig. 7 shows representative examples of mutational signatures in specific tumour entities. Bars show median values, dots show individual values and whiskers show maximum and minimum.

Mutational signatures, reflecting mutational processes of both exogenous and endogenous origins, have been previously characterized for most cancer types^33^. Across all entities, AC2 and AC13, two base substitution signatures that are closely associated to each other and linked with the activation of AID/APOBEC cytidine deaminases, were more pronounced in tumours with chromothripsis (*p* = 0.007 and *p* = 9 × 10^−6^, respectively, Wilcoxon tests; see Fig. 7). Signature 3, the canonical double-strand break signature linked to mutations in *BRCA1* or *BRCA2* or to a BRCAness phenotype, was more pronounced in cases with chromothripsis in liposarcoma (Supplementary Fig. 7). In addition to base substitution mutational signatures, we also analysed small insertion and deletion (ID) mutational signatures. ID2, which is elevated in cancer samples with defective DNA mismatch repair, and ID9 (of unknown aetiology) were significantly more pronounced in tumours with chromothripsis across all entities (*p* = 1.3 × 10^−4^ and 1.5 × 10^−4^, respectively, Wilcoxon tests). Importantly, these differences between mutational signatures detected in tumours with or without chromothripsis were not simply due to differences in the mutational burden (SNVs or indels, respectively), as there was no significant difference in the overall SNV or indel count for these tumour entities. Altogether, prevailing mutational signatures in tumours with chromothripsis may shed light on potential internal and external triggers for chromothripsis.

### Longitudinal analyses suggest clonal heterogeneity

**Fig. 8:**
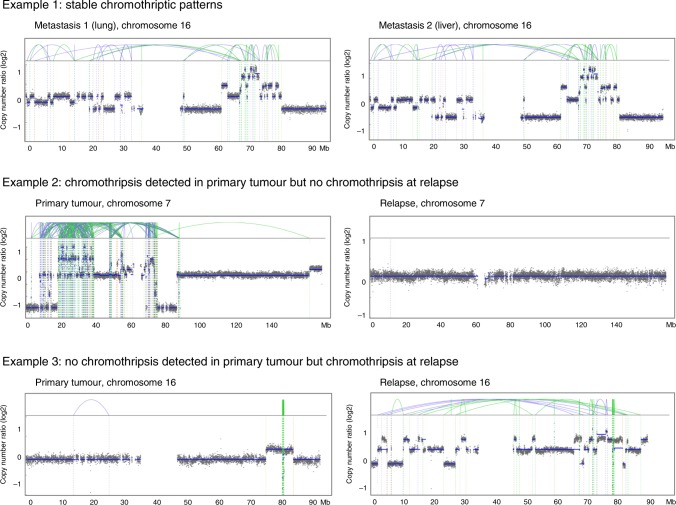
Longitudinal analysis of chromothriptic patterns in matched primary-relapse pairs and different metastases from the same patients. Representative examples of the evolution of chromothriptic patterns are shown. Analyses of the evolution of chromothriptic patterns for all matched pairs (*n* = 41, with 24 pairs showing chromothripsis) are shown in Supplementary Data 5. Example 2 is from a different patient cohort because examples for this specific scenario with available whole-genome sequences for both time points were lacking. Blue lines indicate inversions, green lines indicate break ends, brown lines indicate translocations and orange lines indicate copy-number variation.

Chromothripsis is typically described as an early causative event in tumour development^1,2^. This implies that the chromothriptic chromosome should be detectable in the vast majority of the tumour cells, if chromothripsis generates drivers conferring a selective advantage. However, we and others reported cases with different chromothriptic patterns between matched primary and relapsed tumours, suggesting subclonal evolution^20,34^. We systematically analysed the longitudinal evolution of chromothriptic chromosomes for 24 matched pairs including primary and relapsed tumours but also metastases to different sites (41 pairs in total, including 17 pairs without chromothripsis). Three types of scenarios were observed (Fig. 8 and Supplementary Data 5). First, we detected cases with stable chromothriptic patterns between primary and relapse, showing similar profiles, with chromothripsis detected on the same chromosomes. This scenario supports the common view of chromothripsis as an early event, presuming the presence of the chromothriptic chromosome in the vast majority of the tumour cells. Surprisingly, we observed this first scenario with stable chromothriptic patterns in only half of the matched tumour pairs. As a second scenario, we observed cases for which chromothripsis was detected in the major clone in the primary tumour, but undetectable at relapse. This elimination of the chromothriptic clone suggests a higher sensitivity to treatment for the clone with the chromothriptic chromosome. Finally, we observed cases for which chromothripsis was not detectable in the primary tumour, but detected at relapse. This scenario would imply either the presence of a small clone with chromothripsis undetected at the first time point (clone size below the detection limit or high intra-tumour spatial heterogeneity), or later spontaneous or therapy-induced chromothripsis. Importantly, the tumour content was high (see Supplementary data 5), excluding that chromothripsis in the major clone could have been missed. Fingerprinting eliminated any sample swap, and SNV analysis excluded rare cases for which the first and second tumours were independent cancers arising in the same individual. Altogether, longitudinal analyses revealed important insights related to basic biological processes (e.g., clonality and selection in tumours with chromothripsis) but also have putative therapeutic implications with respect to possible therapy-induced chromothripsis and intra-tumour heterogeneity.

## Discussion

Our comprehensive analysis of chromothripsis in cancer showed a considerably higher prevalence of this phenomenon as compared to initial estimates, with 49% of all cases showing chromothripsis. Despite particularities of this pan-cancer cohort outlined above, this discrepancy reveals an under-estimation of chromothriptic events when using scoring on array-based data. Chromothripsis likely initiates a substantial proportion of human cancers. Importantly, another pan-cancer study not focusing on advanced disease patients, with no enrichment for tumour entities such as sarcomas (known to show a high chromothripsis prevalence) and with no age selection reported chromothripsis prevalence rates in the same range^15^. For instance, the authors described a prevalence of 77% in osteosarcoma (71% in our cohort) and 40% in lung carcinoma (33.3% in our cohort). In a few instances, such as liver adenocarcinoma, the differences in the proportions of tumours with chromothripsis were substantial between both studies (<25 versus 85%), possibly due to different molecular subgroups of patients in both cohorts.

To ensure maximal comparability between tumour entities and between studies, it is essential to apply consistent and well-defined criteria to score for chromothripsis^3^. As we used the same sequencing workflow and the same scoring criteria for all cases in this cohort, we were able to identify tissue types that are more or less prone to chromothriptic events, respectively. For instance, we detected chromothripsis in all cases of malignant peripheral nerve sheath tumour (*n* = 6). In the pancreas, we detected a high chromothripsis prevalence in pancreas carcinomas (61.5%, *n* = 13) but a low prevalence in pancreatic neuroendocrine tumours (15.4%, *n* = 13). This finding suggests different predispositions to chromothriptic events between cell types within one tissue, possibly linked to differences in sensitivity to replication stress, proliferation rate and efficiency of the apoptotic response.

We observed marked differences in chromothriptic patterns between tumour entities, regarding the prevalence of canonical versus non-canonical chromothripsis, involvement of the telomere or centromere in the chromothriptic region, or the number of chromothriptic chromosomes per tumour. This variety of patterns suggests different mechanisms leading to chromothripsis across tumour entities. Entity-specific links between chromothripsis and distinct mutational signatures (e.g., APOBEC activity, homologous recombination repair deficiency) may help deciphering the mechanisms leading to chromothripsis in each tumour entity.

In each tumour type, chromothripsis affected different chromosomes, likely due to a selection for entity-specific driver genes (even though we cannot exclude the role of tissue-specific chromosome territories). Interestingly, chromothriptic events generated a substantial proportion of fusion genes, which might have important clinical implications, as fusion genes provide attractive therapeutic targets. Fusion genes generated due to chromothriptic events were reported recently in lung cancer in non-smokers^35^. Our study showed that this is a general phenomenon, which plays a role across most tumour entities.

Importantly, we analysed the longitudinal evolution of chromothriptic patterns in the largest cohort of matched tumour pairs to date (*n* = 24 pairs with chromothripsis). This analysis revealed that stable chromothriptic patterns between different tumours of the same patient do not represent the prevailing scenario. For a substantial proportion of cases, we detected chromothripsis in the primary tumour, but not in the major clone at relapse, or conversely, only at relapse, but not in the primary tumour. Conceptually, this suggests that the view of chromothripsis as a single early event, with clonal detection of the chromothriptic chromosome, only holds true in a subset of cases. In addition, this finding has potential treatment implications, because targets related to chromothripsis will not necessarily be ubiquitous. Furthermore, potential therapy-induced chromothriptic events or therapies leading to the selection of chromothriptic clones may complicate therapeutic intervention.

Dissecting the commonalities and differences of chromothriptic patterns across tumour entities will lead to a better understanding of chromothripsis in cancer, and potentially lay the basis for the development of novel strategies to target tumour cells.

## Methods

### Study design and participants

The whole-genome and whole-exome sequencing data were generated within the NCT/DKTK MASTER program^16^, a registry trial and analytical platform for prospective, omics-driven stratification of younger adults with advanced-stage cancers across all histologies and patients with rare tumours. Tumour tissue and matched normal control sample for sequencing (from the patient’s whole blood or leukocyte pellet) were obtained after receiving a written informed consent under an institutional review board-approved protocol.

### Genome alignment and variant calling

Whole-genome sequencing data and whole-exome sequencing data were processed by the DKFZ OTP pipeline^36^. The pipeline used BWA-MEM (v0.7.15) for alignment, biobambam (https://github.com/gt1/biobambam) for sorting and sambamba for marking duplication. The tumour-germline paired alignments were then fed to DKFZ indel SNV callers for indel and SNV discovery, as described previously^37^.

### Structural variants and copy number calling

We performed copy-number analysis and structural variant calling from whole-genome sequencing data. Two structural variant callers, SvABA v134^38^ and SOPHIA v1.2.16 (https://bitbucket.org/utoprak/sophia/src) were used. SvABA is a structural variant caller based on assembly and discordant read based approach. SOPHIA is a structural variant caller based on the supplementary alignment approach. SOPHIA is integrated in the DKFZ OTP pipeline, where the output was used in combination with alignment files for ploidy estimation and copy number calling using ACESeq v1.2.8^39^. SvABA outputs were used for structural variant calling for the analysis of microhomologies at the breakpoints.

### Copy number analysis from whole-exome sequencing data

Copy number analysis from whole-exome sequencing data was performed by EXCAVATOR2^40^, which allows hybrid bin size on captured regions and off-target regions (reads available from the sequencing data but not located in exonic regions). The captured regions were plotted as deep blue and off-target regions were represented by cyan colour by the plotting function of EXCAVATOR2 (see Supplementary Fig. 1). All regions were used for copy number segmentation and copy number calling.

### Inference of chromothripsis by visual scoring

For visual evaluation of chromothripsis status, the number of switches between copy-number states was counted for each chromosome. Chromosomes containing 10 or more such switches within 50 Mb were scored as chromothripsis-positive with high confidence. Chromosomes with 8 to 9 or 6 to 7 switches within 50 Mb were scored as chromothripsis-positive with intermediate and low confidence, respectively. Within identified chromothripsis-positive regions, the number of distinct copy-number states was counted, and the involvement of telomere or centromere within the chromothriptic region was assessed.

### Inference of chromothripsis by algorithm-based scoring

In silico chromothripsis scoring was performed by Shatterseek^12^. Copy number variants from ACESeq (https://github.com/DKFZ-ODCF/ACEseqWorkflow) and structural variants from SOPHIA were used as input. We applied the same criteria as previous studies to define a positive call^3^.

### Quantification of indel signatures and indel calling

Indels were called by two software tools, platypus^41^ and Mutect2^42^. The output of the two tools were intersected to produce a combined set of high-confidence indels. The combined set was further filtered by an in-house blacklist of indels to remove artefacts. The filtered output was converted into 83 indel subclasses by the PCAWG signature preparation tool^43^. Finally, the Indel exposures were estimated by SigProfiler (v2.5.1)^43^ on each tumour by indel signatures defined by COSMIC signatures V3^43^.

### Quantification of SNV mutational signatures

Somatic high-confidence SNVs were used as input for YAPSA^44^ to perform a mutational signature analysis and retrieve the exposure of 30 SNV signatures from COSMIC V2 (https://cancer.sanger.ac.uk/cosmic/signatures_v2).

### Identification of fusion genes

Fusion genes from the RNA-seq data were identified by Arriba (Arriba: Fast and accurate gene fusion detection from RNA-seq data, https://github.com/suhrig/arriba). Candidate fusions from medium and high confidence were further validated by analysing structural variants from the whole-genome sequencing identified by SOPHIA. These structural variants called by SOPHIA within 200 kb of fusion calls were combined into a high confidence set. We performed a regression analysis to compare the number of fusions per breakpoint in tumours with chromothripsis as compared to tumours without chromothripsis (see Fig. 4 and Supplementary Data 4).

### Significance of chromothriptic events per chromosome

We evaluated the likelihood of the observed number of chromothripsis events per chromosome per entity (see Supplementary data 3). Tumour entities with more than 10 cases with positive chromothripsis scoring were selected for permutation analysis. Random and non-overlapping regions were sampled from chromosome 1 to chromosome X. Size of the resampled regions are identical to the size of the chromothriptic regions per tumour. Resampling was performed 50000 times, evaluating the amount of random samples per chromosome. The total number of successes is counted as the peak number of events per chromosome exceeding or equal to the observed peak of chromothriptic events.

### Analysis of telomere features

For whole-genome sequenced cases (*n* = 316), telomere characteristics were characterized by TelomereHunter^45^ (1.1.0). Ten telomere variant repeats including TCAGGG, TGAGGG, TTGGGG, TTCGGG, TTTGGG, ATAGGG, CATGGG, CTAGGG, GTAGGG and TAAGGG were quantified and log2 ratio between tumour and germline specimens were computed. SNV calling identified truncating mutations in *ATRX* and *DAXX* as well as *TERT* promoter mutations.

### Connecting chromothriptic chromosomes with *TERT* gains

To evaluate if *TERT* gains are connected to chromothriptic regions by structural variants, genome walking was performed on structural variants upstream and downstream of *TERT*. At most two upstream and two downstream structural variants of *TERT* were followed and the walking was restricted to a walking distance of 50 Mb. Each connection was overlapped with the chromothriptic regions to evaluate the prevalence of such links. Chromothriptic cases with *TERT* connected to chromothriptic regions were contrasted to chromothriptic cases without connection to *TERT*, by the proportions of *TERT* gain using chi-square test.

### Microhomologies at the breakpoints and DNA repair processes

Structural variants were called by SvaBA^46^, an assembly and discordant read based approach for structural variants discovery. The HOMO field was retrieved for each structural variant called by the assembly method of SvABA. To estimate the contribution of different homology sizes, the structural variants with homology information were binned for analysis and visualization. There are 5 bins for homology usage: blunt end to 1, 2, 3–5, 6–9, and >10 bp. The proportions of each bin were normalized by the total number of structural variants, where significance was assessed by beta-regression.

### Pathogenic germline variants in cancer predisposition genes

SNVs and indels were called in the tumour sample and subsequently annotated as germline variants in case they were detected in the control sample derived from the patient’s whole blood or leukocyte pellet. Rare germline SNVs and indels in a list of cancer predisposition genes were filtered and assessed according to the AMP-ACMG guidelines.

### Statistical analysis

Statistical analyses and visualizations were performed using R^47^ and ggplot2^48^. For comparison of mutational signatures, Wilcoxon rank-sum test was applied on log2 absolute exposures for statistical testing. The significance for the telomere repeats between groups were assessed by *t* test on the log2 values between tumour and germline on each telomeric repeats and on total telomere content. Family-wise correction of *p* values was performed according to Bonferroni on statistic contrasting mutational signatures, microhomologies, chromothripsis occurrence per chromosome, and telomere content.

### Reporting summary

Further information on research design is available in the Nature Research Reporting Summary linked to this article.

## Supplementary information

Supplementary Information

Description of Additional Supplementary Files

Supplementary Data 1

Supplementary Data 2

Supplementary Data 3

Supplementary Data 4

Supplementary Data 5

Reporting Summary

## Data Availability

Sequencing data were deposited in the European Genome-phenome Archive under accession number EGAS00001004250. All other data is available within the Article, Supplementary Information or available from the authors upon reasonable request. https://ega-archive.org/studies/EGAS00001004250
