# Supplementary material for: The landscape of chromothripsis across adult cancer types
Source: Nat Commun. 2020 May 8;11:2320. doi: 10.1038/s41467-020-16134-7 (PMC7210959; doi:10.1038/s41467-020-16134-7)
Supplement: Supplementary file 1 — Supplementary Information [file 41467_2020_16134_MOESM1_ESM.pdf]

Supplementary Information

The landscape of chromothripsis across adult cancer types

Voronina & Wong et al

Supplementary Table 1. Structural variants between *TERT* locus and chromothriptic chromosomes

	TERT duplicated	*TERT* not duplicated
Chromothripsis-positive cases, chromothriptic region **linked with *TERT***	12	5
Chromothripsis-positive cases, chromothriptic region **not linked with *TERT***	47	71
		**Chi-sq p <= 0.01683**

Total cases with chromothripsis	152
Chromothripsis-positive cases, chromothriptic region overlapped with *TERT*	17
Chromothriptic region not overlapped with *TERT* , but linked	17
Chromothriptic region not overlapped with *TERT* and not linked	118

Supplementary Figure 1

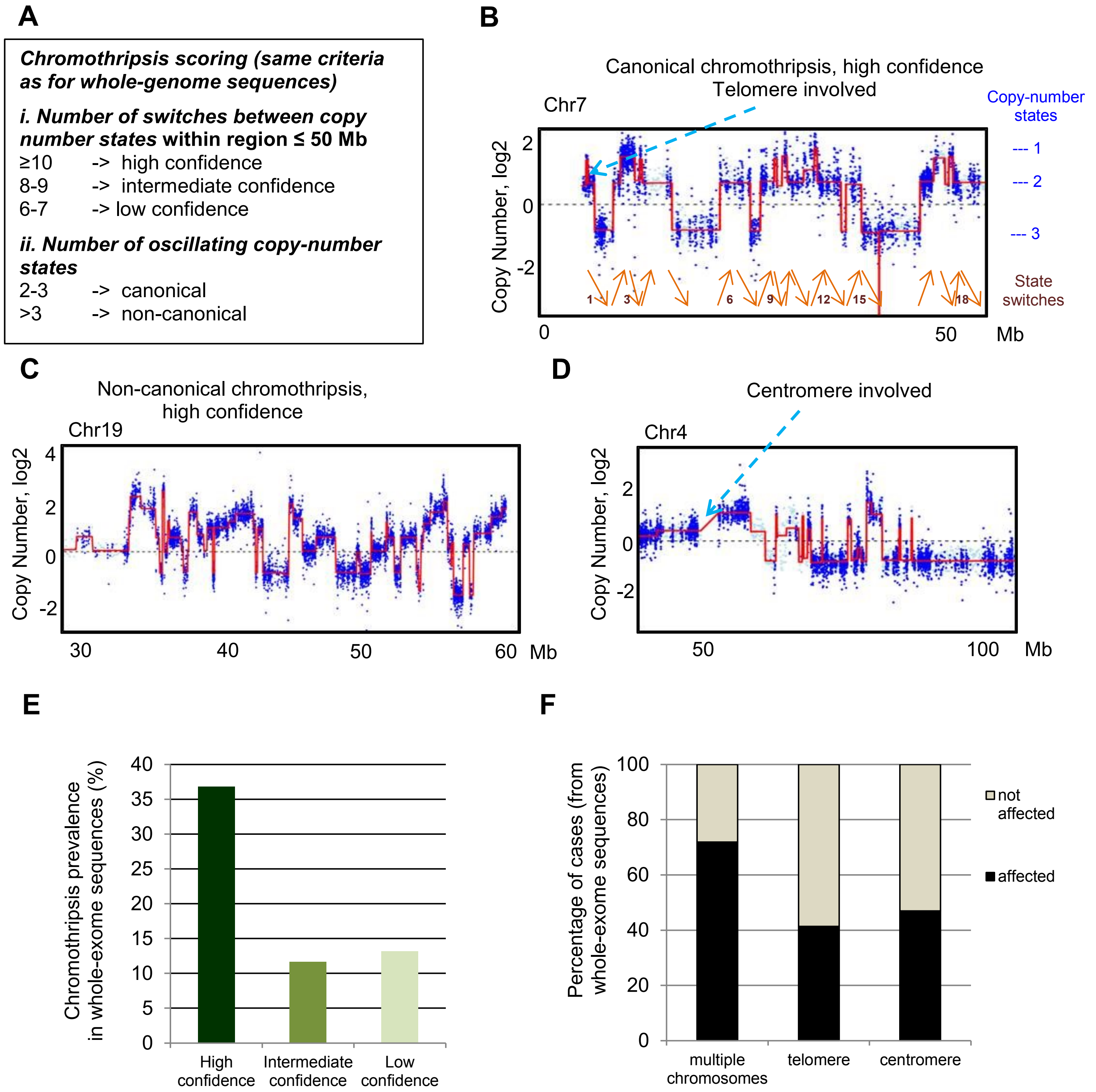

Supplementary Figure 1. Chromothriptic patterns and prevalence, scoring based on whole-exome sequences. **A**. Chromothripsis scoring: criteria to determine the confidence of the scoring and to define canonical versus non-canonical chromothripsis. **B**. Representative whole-exome sequenced example of canonical chromothripsis. In this case, the telomere is included in the chromothriptic region **C**. Representative whole-exome sequenced example of non-canonical chromothripsis. **D**. Representative whole-exome sequenced example of a chromothriptic chromosome for which the centromere region is involved. **E**. Chromothripsis prevalence shown as percentage of cases (n=318, whole-exome sequencing) including cases with high confidence, intermediate confidence and low confidence chromothripsis. **F**. Whole-exome sequenced chromothripsis cases with multiple versus single chromosomes affected; with or without telomere involvement (illustrated in B), as well as with or without centromere involvement (illustrated in D).

Supplementary Figure 2

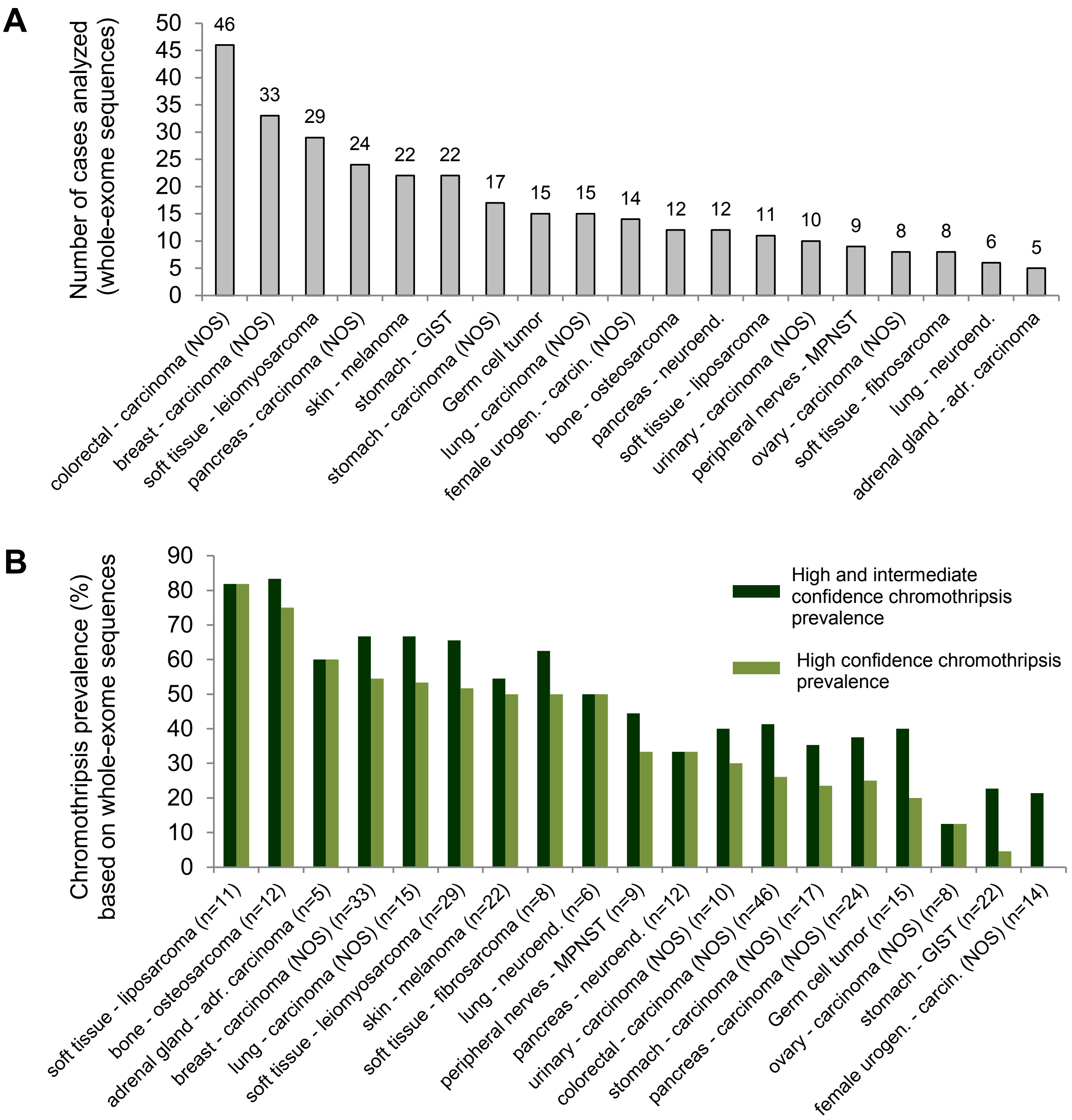

Supplementary Figure 2. Overview of the tumour entities represented in the cohort with whole-exome sequencing data and chromothripsis prevalence across entities. **A.** Number of whole-exome sequenced cases for each tumour entity (n=318 in total, whole-exome sequences) **B.** Chromothripsis prevalence (high confidence, light green; high and intermediate confidence, dark green) per tumour entity for all entities with more than 3 whole-exome sequenced cases (n=318). MPNST, malignant peripheral nerve sheath tumor; GIST, gastrointestinal stromal tumor; NOS, not otherwise specified.

Supplementary Figure 3

Chromothripsis occurrence frequency per chromosome
Whole-genome sequencing data

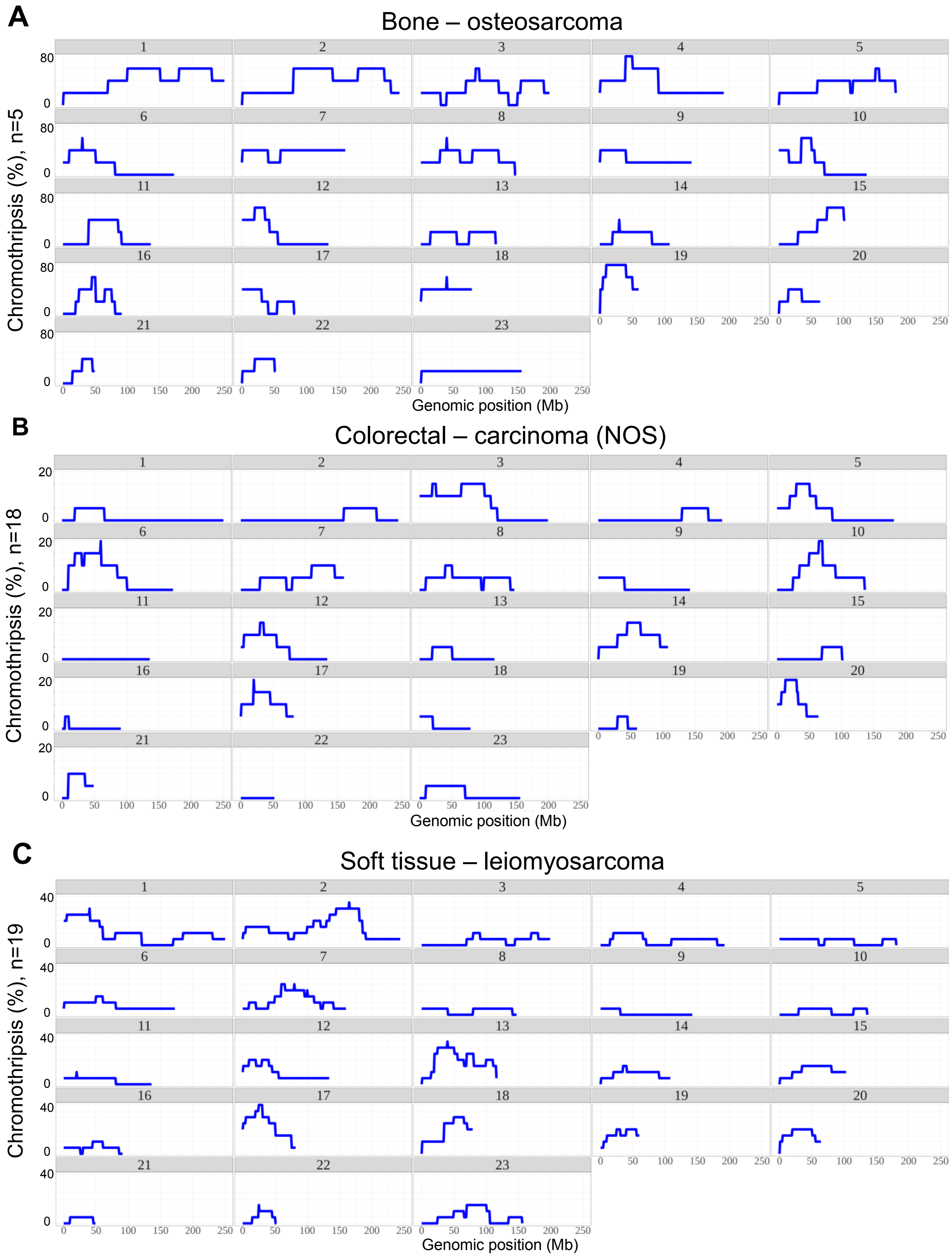

Supplementary Figure 3

D

Soft tissue – liposarcoma

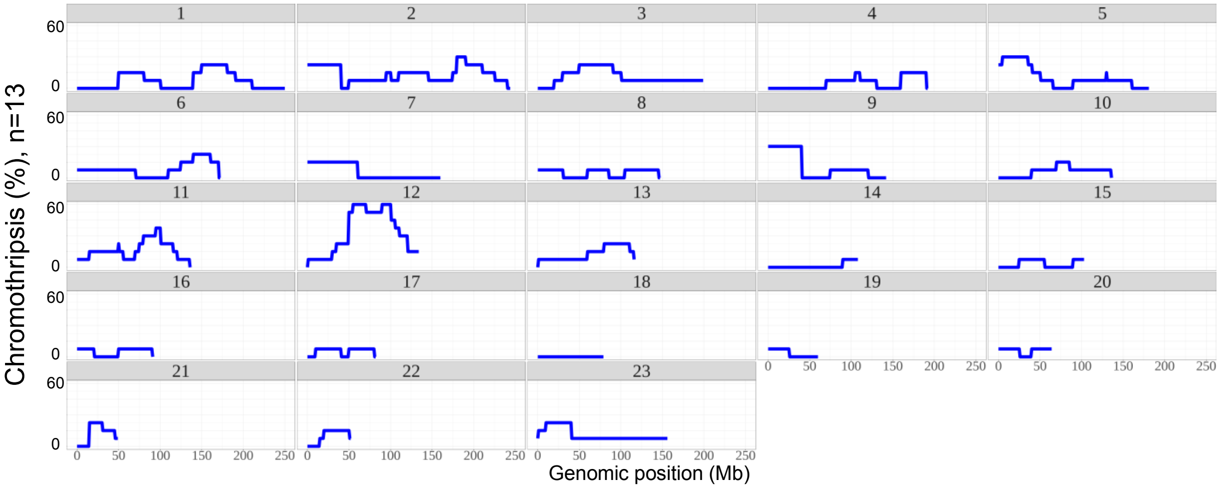

Chromothripsis occurrence frequency per chromosome
Whole-genome and whole-exome sequencing data combined

E

Adrenal gland – adrenocortical carcinoma

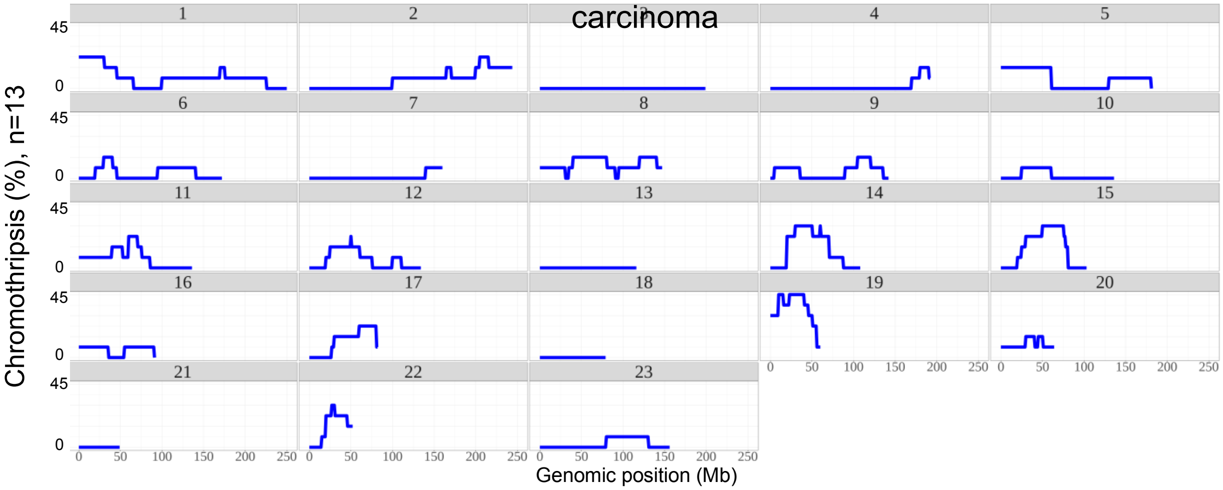

F

Bone – osteosarcoma

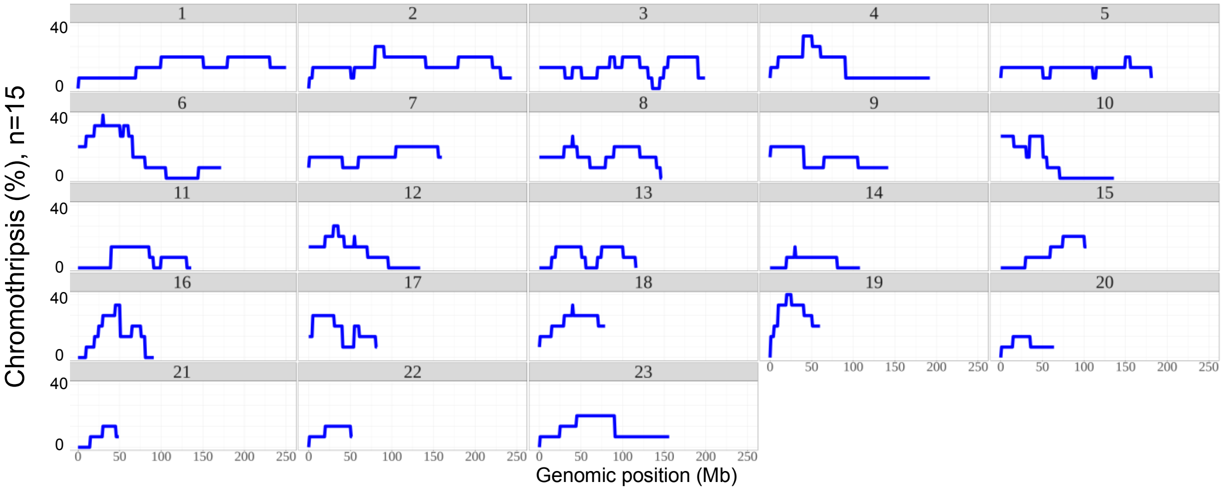

Supplementary Figure 3

G

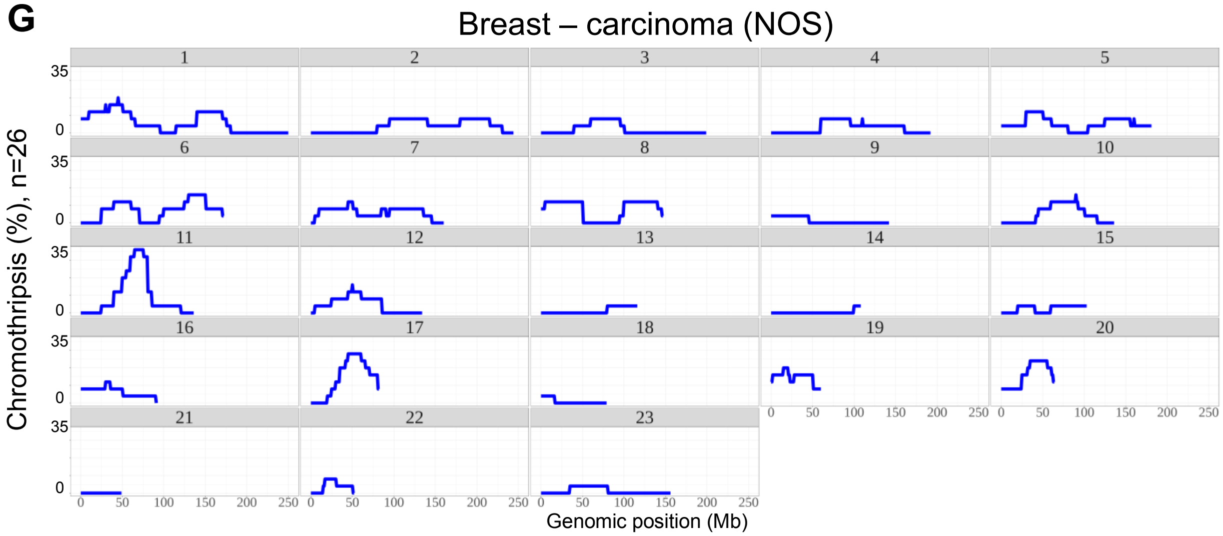

H

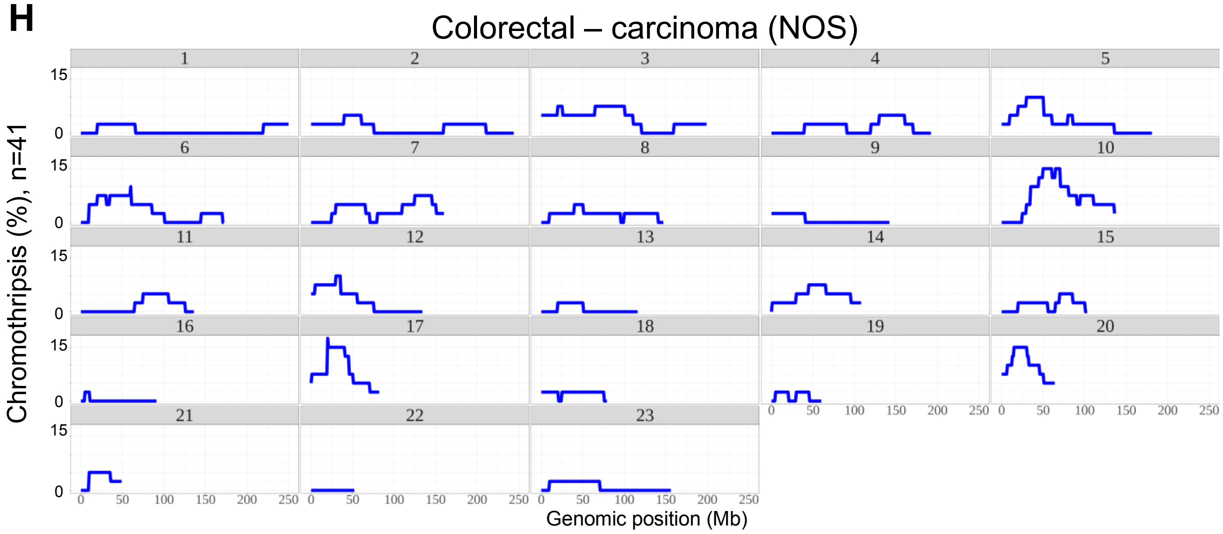

I

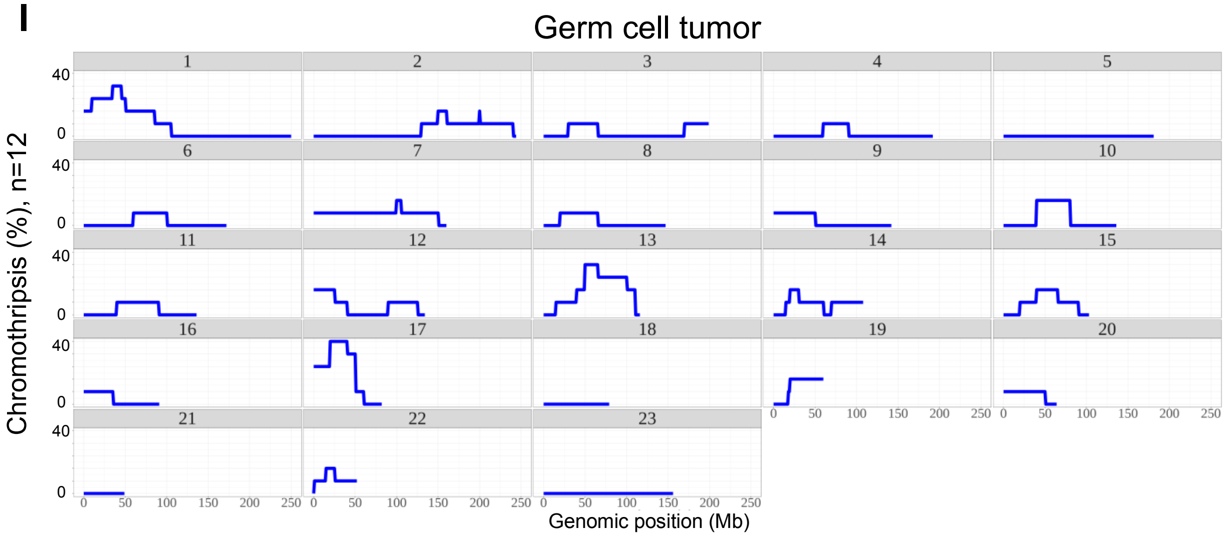

Supplementary Figure 3

J

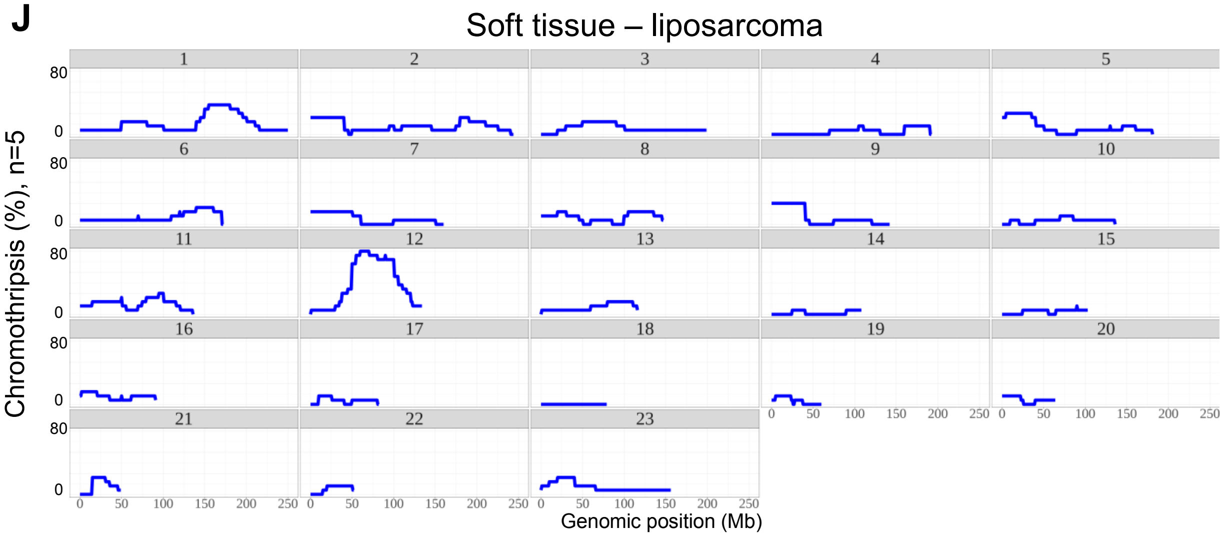

K

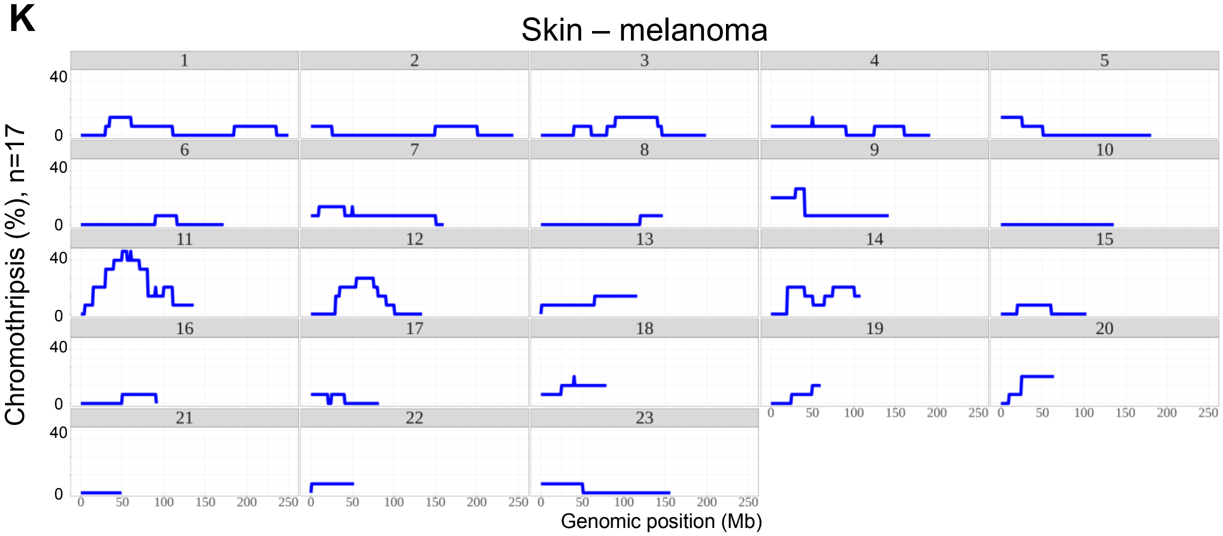

L

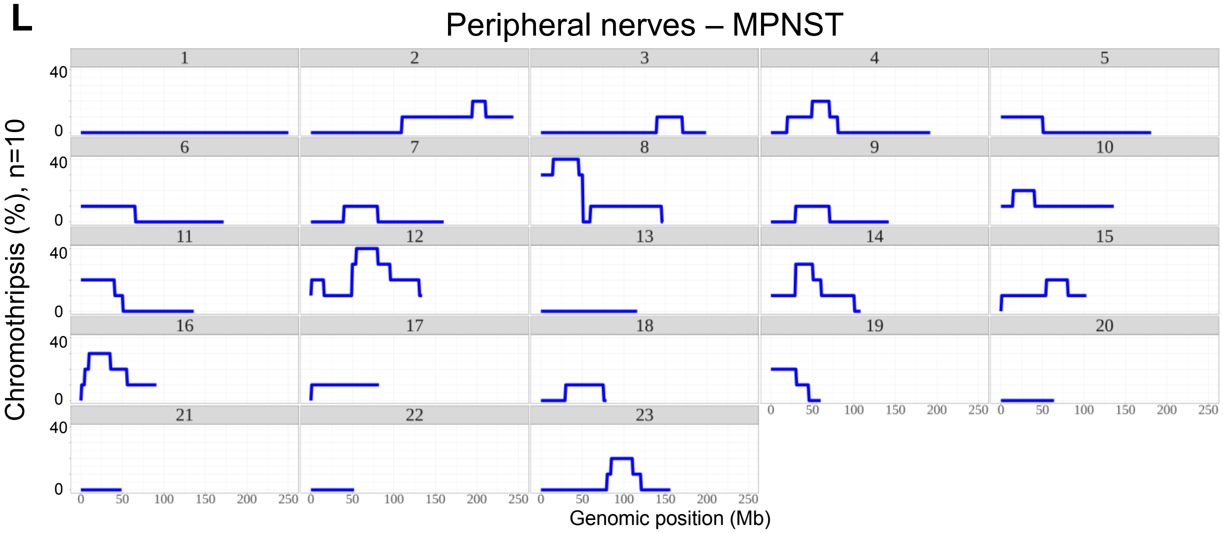

Supplementary Figure 3

M

Stomach – carcinoma (NOS)

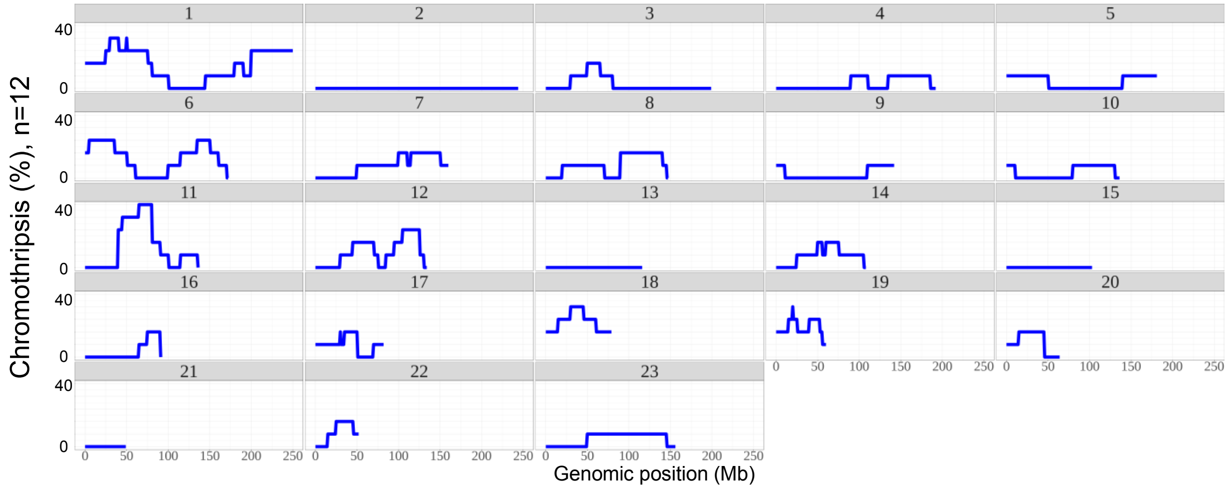

Copy number changes per chromosome in chromothriptic versus non-chromothriptic cases
Whole-genome sequencing data

N

Adrenal grand – adrenal carcinoma

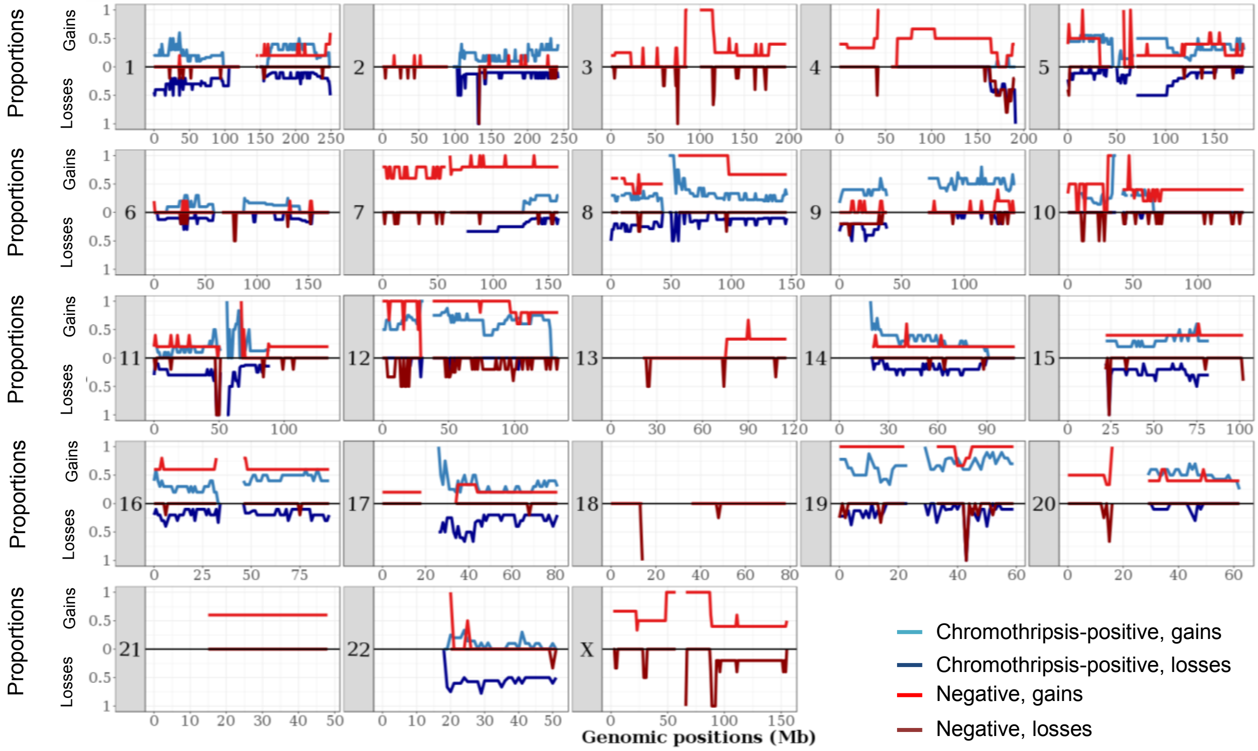

Supplementary Figure 3

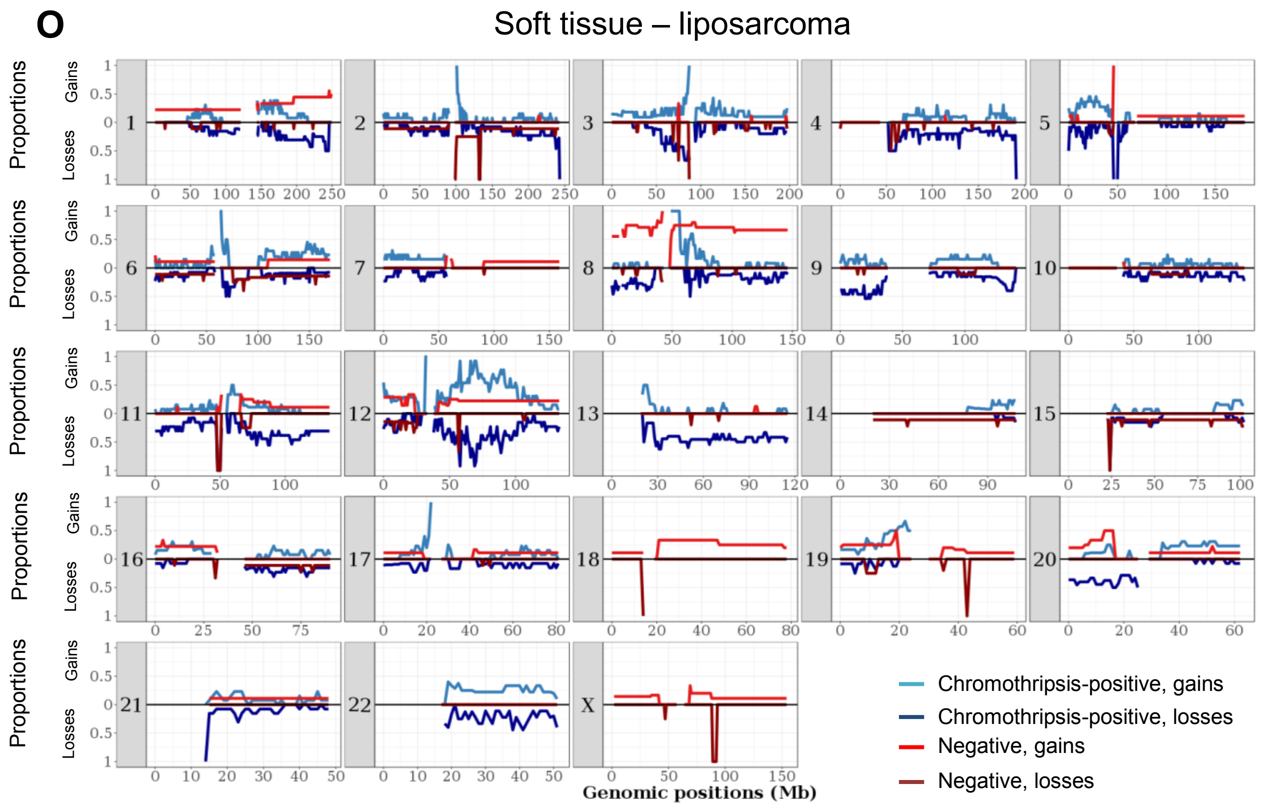

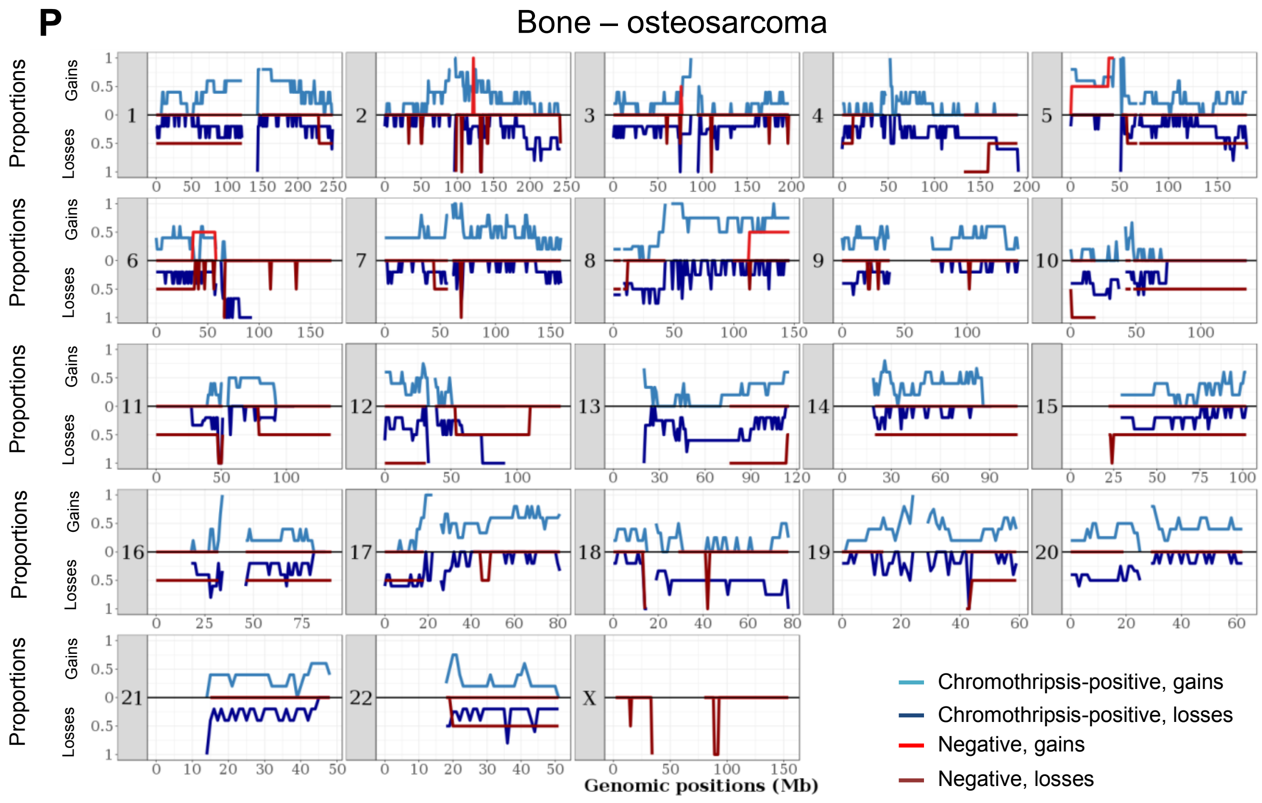

Supplementary Figure 3

Q Colorectal – Carcinoma (NOS)

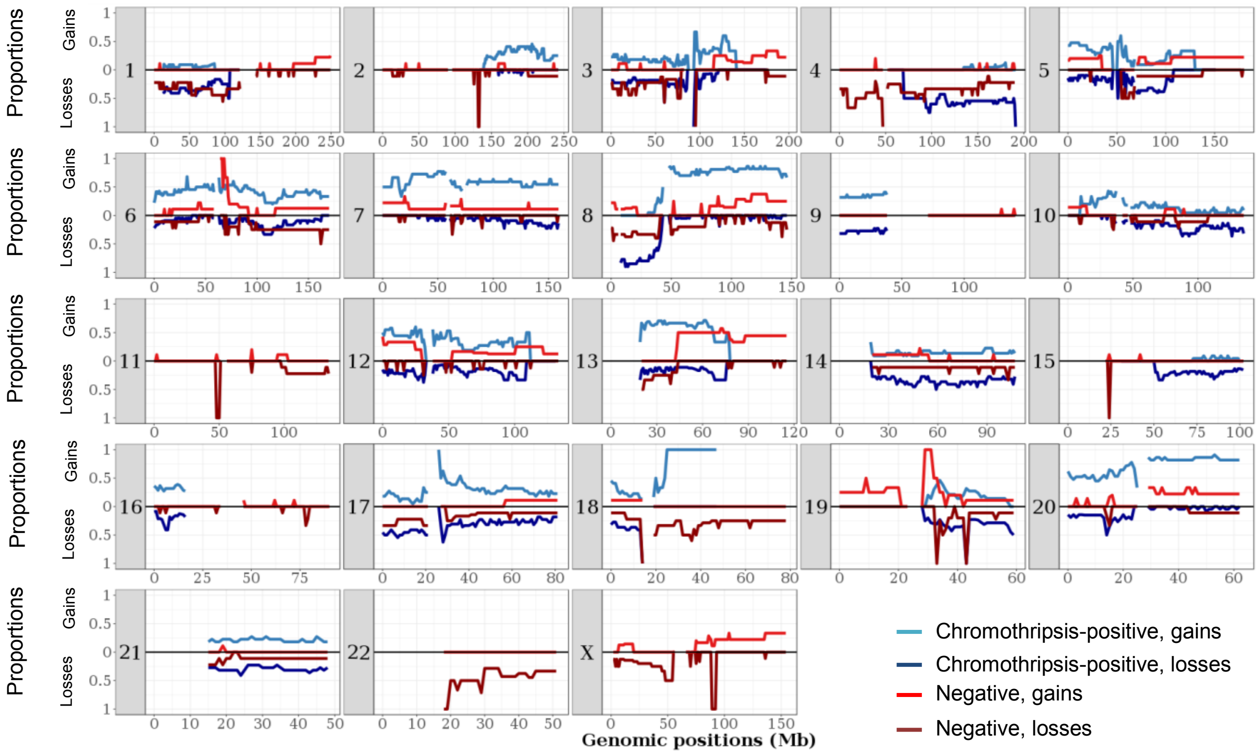

R Soft tissue – leiomyosarcoma

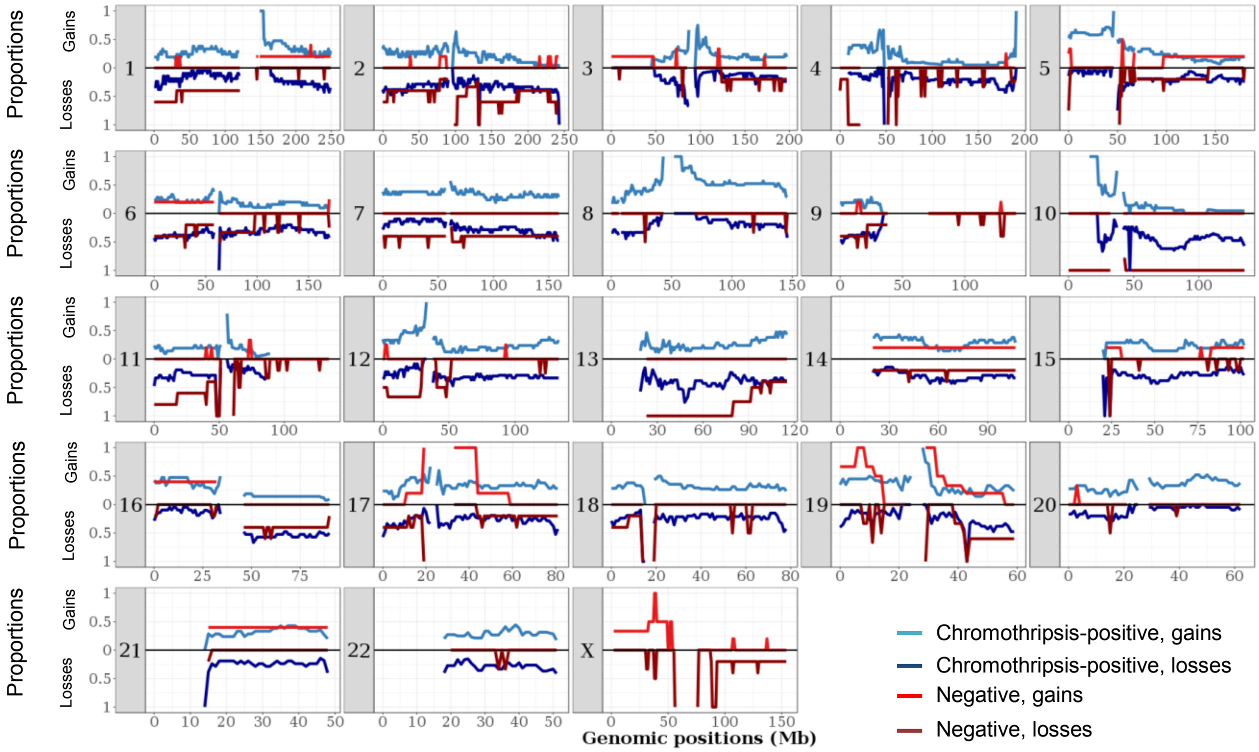

Supplementary Figure 3

Supplementary Figure 3. Frequency of chromothriptic events across chromosomes for various tumour entities. Panels A-D represent chromothripsis scoring results based on whole-genome sequencing for these entities where number of cases was sufficient to detect frequently affected with chromothripsis chromosomes. Panels E-M reflect combined results of both whole-genome and whole-exome sequencing scoring. The total number of high and intermediate confidence chromothripsis-positive cases is indicated as n. The Y axis shows the percentage of chromothriptic events affecting each chromosomal fragment from all chromothriptic cases. Panels N-R demonstrate the proportions of gains (upper panels) and losses (lower panels) in tumours with (blue) or without chromothripsis (red) for entities for which the number of cases in both groups (with chromothripsis or without chromothripsis) is high enough to plot the combined frequencies of gains and losses, respectively. Abbreviations: MPNST, malignant peripheral nerve sheath tumour; NOS – not otherwise specified.

Supplementary Figure 4

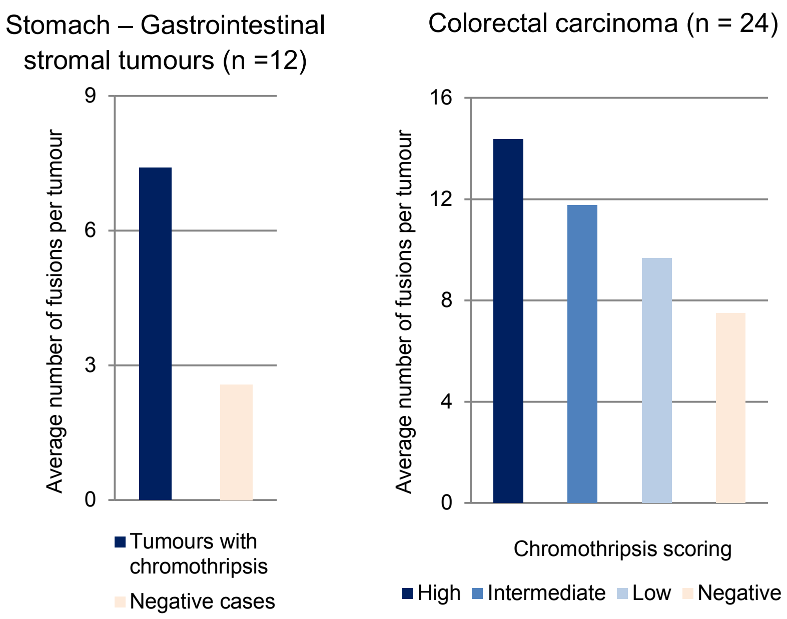

Supplementary Figure 4. Enrichment for gene fusions on chromothriptic chromosomes. Enrichment is illustrated in two selected tumour entities. The difference is not statistically significant when corrected for multiple testing.

Supplementary Figure 5

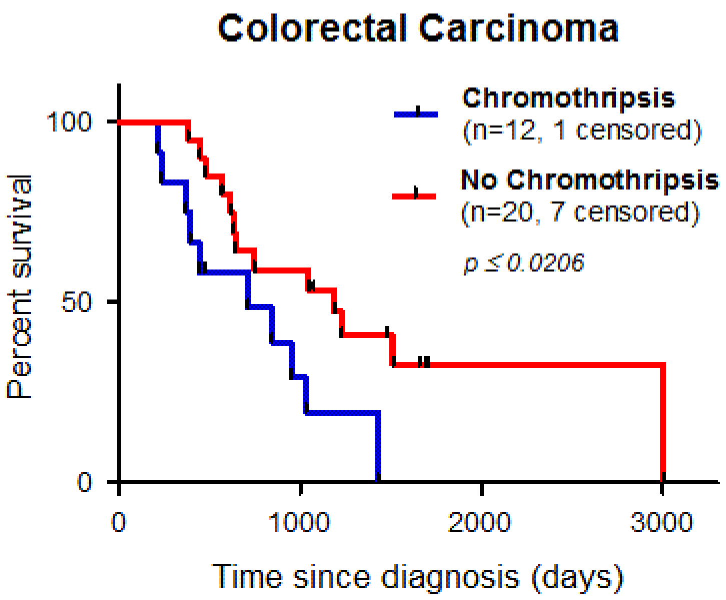

Supplementary Figure 5. Chromothripsis and clinical outcome (overall survival). Kaplan-Meier plot for patients with colorectal carcinoma. Chromothripsis scoring was done based on whole-exome sequences. All patients are advanced disease colorectal cancer patients with metastases, median age of the chromothripsis-positive group is 40.7 years, chromothripsis-negative group – 42.5 years. Patients who were still alive at the time of analysis were censored, the total number of available cases for the analysis is indicated as n. Statistical significance was tested using log-rank Mantel-Cox test (two-sided; no multiple testing adjustment of the p-value was necessary as this was the only tumour entity for which this comparison was possible, due to the number of patients in both groups for which survival data were available).

Supplementary Figure 6

Repair process for double-strand breaks	Average number of base pairs of microhomology
Non-homologous end-joining (NHEJ)	0-1 bp *
Alternative end-joining (Alt-EJ)	3-5 bp *
Homologous recombination (HR)	>10 bp *

*For this repair process, this is the average number of base pairs of microhomology observed

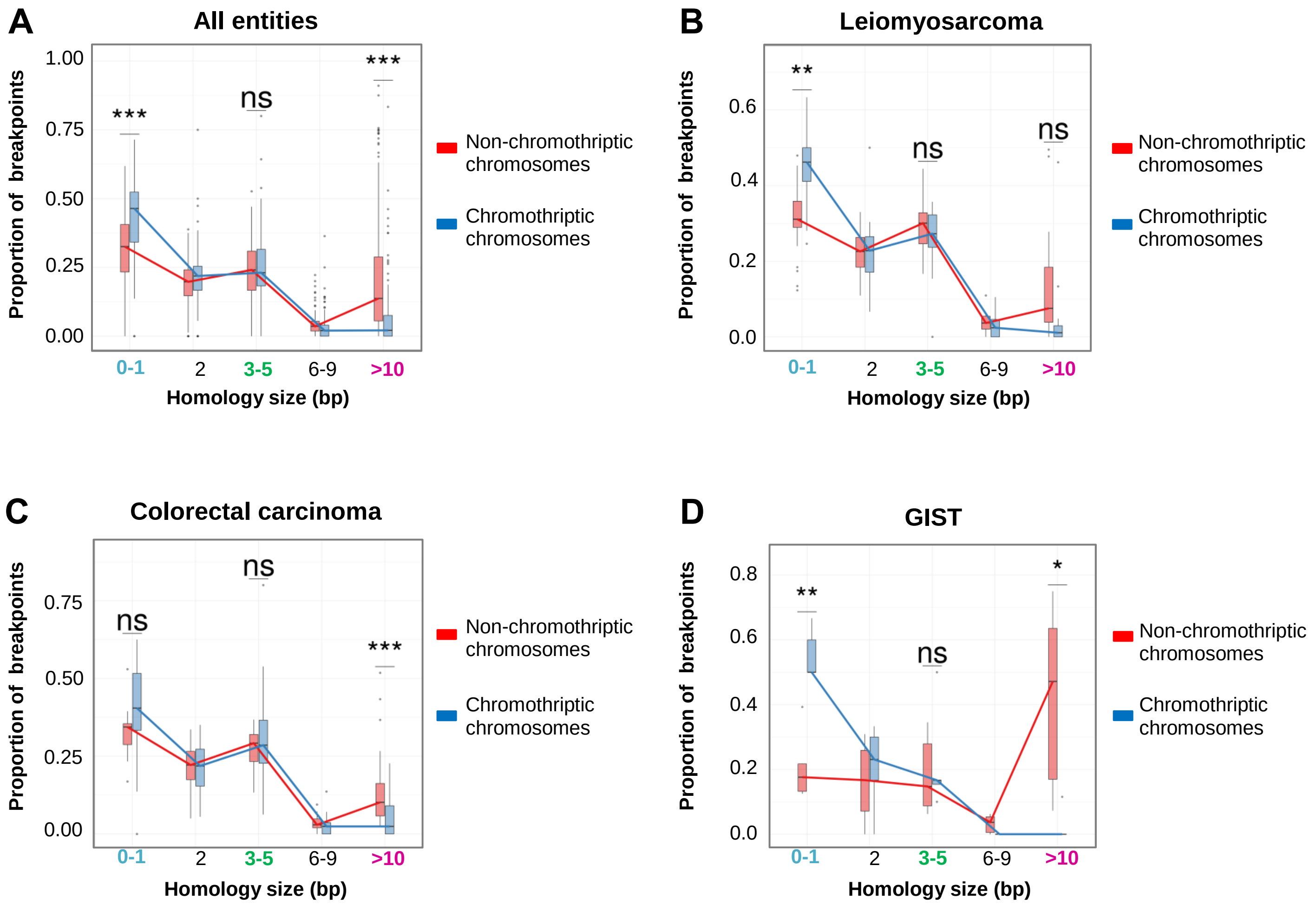

<u>Supplementary Figure 6</u>. Major DNA repair processes involved in the rejoining of the breakpoints after chromothripsis. Based on the number of base pairs of homology at the breakpoint sites, we can infer the prevailing repair processes involved in the rejoining of the DNA fragments. The regional comparison was carried out: chromothriptic chromosomes were analyzed vs non-chromothriptic chromosomes. Centre lines show median values, bounds of boxes show 75th percentiles and whiskers show maximum and minimum. (*, $p < 0.05$; **, $p < 0.01$; ***, $p < 0.001$). Statistical significance was tested using beta-regression analyses and Bonferroni correction applied for multiple testing correction. Abbreviations: GIST – gastrointestinal stromal tumours.

Supplementary Figure 7

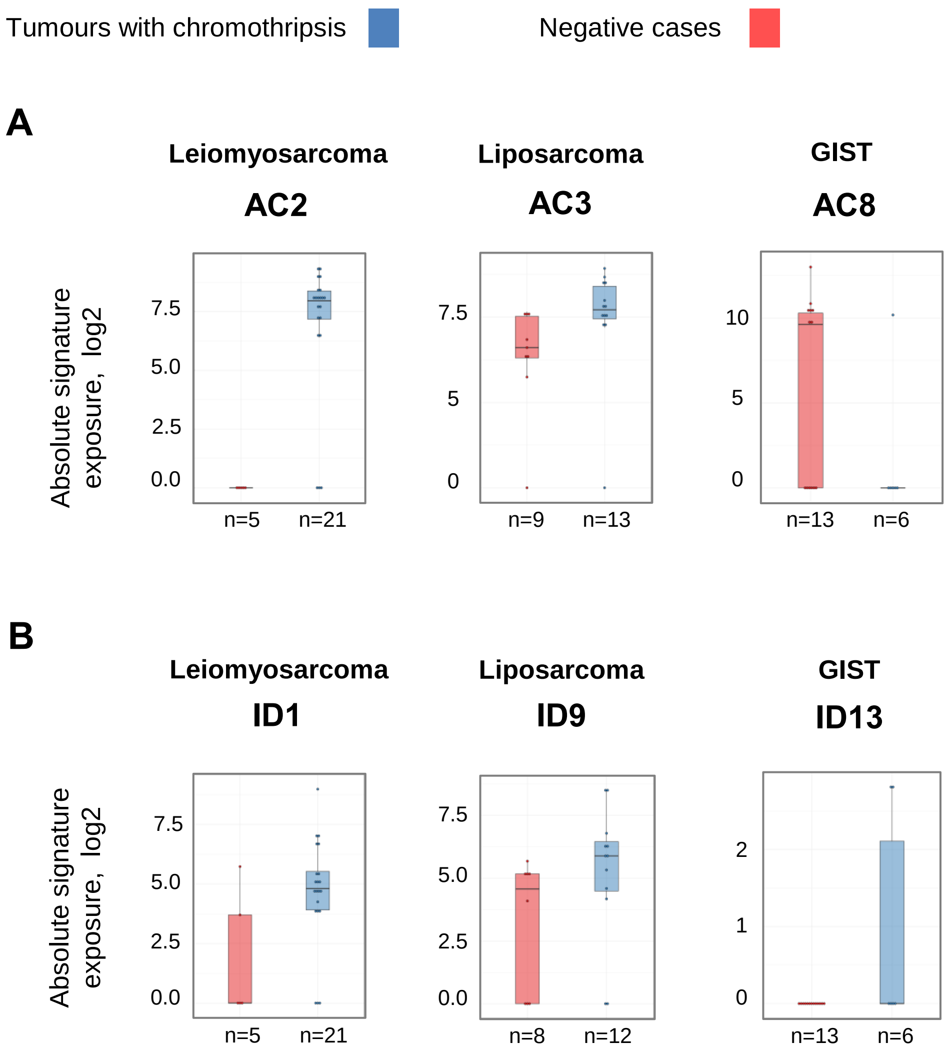

Supplementary Figure 7. Chromothripsis is linked with specific mutational signatures. Representative examples of base substitution signatures **(A)** and indel signatures **(B)** in chromothripsis-positive versus chromothripsis-negative tumours. Bars show median values, dots show individual values and whiskers show maximum and minimum. Significance was tested using Wilcoxon tests and Bonferroni correction for the multiple testing (two-sided, the cut-off criteria for analysed entities included at least 5 tumours in the positive group as well as in the negative group and at least 5 non-zero signature exposures in the set). Abbreviations: GIST, gastrointestinal stromal tumours.
