# Supplementary material for: The landscape of chromothripsis across adult cancer types
Source: Nat Commun. 2020 May 8;11:2320. doi: 10.1038/s41467-020-16134-7 (PMC7210959; doi:10.1038/s41467-020-16134-7)
Supplement: Supplementary file 2 — Description of Additional Supplementary Files [file 41467_2020_16134_MOESM2_ESM.pdf]

Description of Additional Supplementary Files

File Name: Supplementary Data 1
Description: Description of the cohort, tumour entities and type of available data

File Name: Supplementary Data 2
Description: Chromothripsis inference based on visual scoring and algorithm-based scoring

File Name: Supplementary Data 3
Description: Statistical enrichment of chromothriptic events on specific chromosomes in each tumour entity

File Name: Supplementary Data 4
Description: Fusion genes due to chromothriptic events

File Name: Supplementary Data 5
Description: Longitudinal analysis of chromothriptic patterns in 24 matched pairs
